# Understanding the Mechanical Properties of Ultradeformable Liposomes Using Molecular Dynamics Simulations

**DOI:** 10.1021/acs.jpcb.3c04386

**Published:** 2023-10-25

**Authors:** Jiaming Xu, Vyshnavi Karra, Danielle E. Large, Debra T. Auguste, Francisco R. Hung

**Affiliations:** † Department of Chemical Engineering, Northeastern University, Boston, Massachusetts 02115, United States; ‡ Department of Bioengineering, Northeastern University, Boston, Massachusetts 02115, United States

## Abstract

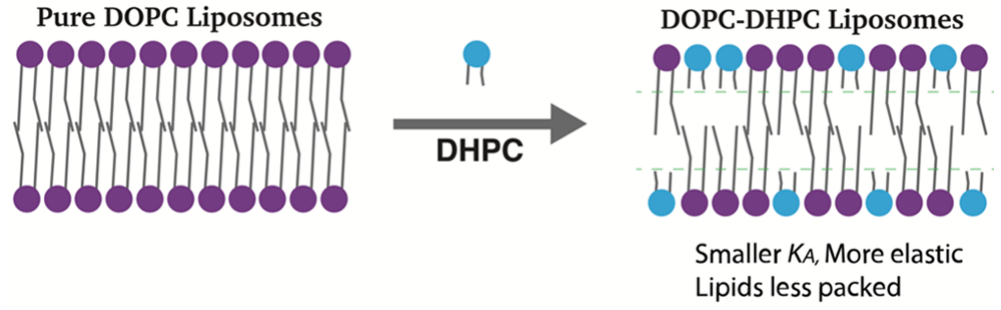

Improving drug delivery efficiency to solid tumor sites is a central challenge in anticancer therapeutic research. Our previous experimental study (Guo et al., *Nat. Commun.***2018**, *9*, 130) showed that soft, elastic liposomes had increased uptake and accumulation in cancer cells and tumors *in vitro* and *in vivo* respectively, relative to rigid particles. As a first step toward understanding how liposomes’ molecular structure and composition modulates their elasticity, we performed all-atom and coarse-grained classical molecular dynamics (MD) simulations of lipid bilayers formed by mixing a long-tailed unsaturated phospholipid with a short-tailed saturated lipid with the same headgroup. The former types of phospholipids considered were 1,2-dioleoyl-*sn*-glycero-3-phosphocholine (DOPC) and 1,2-dipalmitoleoyl-*sn*-glycero-3-phosphocholine (termed here DPMPC). The shorter saturated lipids examined were 1,2-diheptanoyl-*sn*-glycero-3-phosphocholine (DHPC), 1,2-didecanoyl-*sn*-glycero-3-phosphocholine (DDPC), 1,2-dilauroyl-*sn*-glycero-3-phosphocholine (DLPC), and 1,2-dimyristoyl-*sn*-glycero-3-phosphocholine (DMPC). Several lipid concentrations and surface tensions were considered. Our results show that DOPC or DPMPC systems having 25–35 mol % of the shortest lipids DHPC or DDPC are the least rigid, having area compressibility moduli *K*_*A*_ that are ∼10% smaller than the values observed in pure DOPC or DPMPC bilayers. These results agree with experimental measurements of the stretching modulus and lysis tension in liposomes with the same compositions. These mixed systems also have lower areas per lipid and form more uneven *x*–*y* interfaces with water, the tails of both primary and secondary lipids are more disordered, and the terminal methyl groups in the tails of the long lipid DOPC or DPMPC wriggle more in the vertical direction, compared to pure DOPC or DPMPC bilayers or their mixtures with the longer saturated lipid DLPC or DMPC. These observations confirm our hypothesis that adding increasing concentrations of the short unsaturated lipid DHPC or DDPC to DOPC or DPMPC bilayers alters lipid packing and thus makes the resulting liposomes more elastic and less rigid. No formation of lipid nanodomains was noted in our simulations, and no clear trends were observed in the lateral diffusivities of the lipids as the concentration, type of secondary lipid, and surface tension were varied.

## 1 Introduction

The challenge of improving drug delivery efficiency to solid tumor sites remains a central topic in the field of anticancer therapeutic research. Lipid nanoparticles (LNPs), which consist mainly of lipids, have gained prominence due to their enhanced therapeutic outcomes, biocompatibility, and ability to bolster drug stability, amplify solubility, and facilitate controlled release.^1^ Liposomes, a subset of LNPs, are spherical vesicles comprising one or more lipid bilayers encasing an aqueous core. Other LNPs include solid lipid nanoparticles (SLNs) and nanostructured lipid carriers (NLCs).^1−3^ Nanolipogels (NLGs), which are hybrids of liposomes and hydrogels, consist of lipid bilayers encapsulating a hydrogel core. This unique structure combines the stability and controlled release attributes of hydrogels with the encapsulation effectiveness of liposomes, thereby augmenting drug delivery efficiency, stability, and targeting.^4^ First-generation drug formulations based on LNPs and liposomes (e.g., Abraxane and Doxil) have shown clinical benefits,^5−7^ including the extension of progression-free survival to up to 7 months, with significantly less off-target toxicity relative to that of free drug treatment. However, the clinical effectiveness of liposomal drug encapsulation has not significantly surpassed that of free drug.^8−10^ This limited efficacy stems from three fundamental shortcomings: inadequate accumulation of liposomal drug carriers within the tumor, insufficient or heterogeneous penetration into the tumor tissue, and slow or ineffective drug release from the nanoparticle. Improving tumoral accumulation and penetration of drug delivery vehicles remains an important challenge,^11,12^ especially when desmoplasia is present. The desmoplastic response produces a tumor microenvironment enriched with activated stromal cells (fibroblasts and myofibroblasts), which synthesize collagen and other extracellular matrix (ECM) proteins. This dense and fibrous ECM imparts a physiological barrier to tumor drug delivery, impeding the delivery and diffusion of nanoparticles into the tumor.

To improve the shortcomings mentioned above, research on nanocarriers’ physical characteristics has primarily focused on their size, shape, and surface chemistry. However, the effects of nanoparticle mechanical properties remain poorly understood, although studies focusing on the mechanical properties of nanoparticles have been recently reviewed.^13−19^ In drug delivery studies, elastic nanoparticles with low Young’s moduli (45–71 kPa) have exhibited increased cellular uptake,^20^ prolonged blood circulation,^21−23^ reduced uptake by immune cells,^23^ increased tumor accumulation,^20,24^ and a better ability to access challenging tissue targets^25^ relative to their less elastic counterparts. Soft nanoparticles have demonstrated enhanced extravasation from blood vessels and have the ability to deformably navigate through narrow gaps and pores within the tumor microenvironment, resulting in more efficient tumor accumulation compared to stiffer vesicles.^26^ This body of research suggests that elastic, highly flexible liposomes offer a promising drug delivery platform. In a past experimental study, Guo et al.^20^ investigated the cellular and tumor uptake of nanolipogels (NLGs) with four different elasticities. The NLGs were engineered with an alginate core encapsulated by identical lipid bilayers. The elasticity of those NLGs was tuned by adjusting the calcium concentration in the alginate core. The results showed that softer NLGs had increased uptake by cancer cells and increased penetration in multicellular cancer spheroids *in vitro* as well as increased tumor accumulation in *in vivo* studies, compared to more rigid NLGs. The authors proposed that soft NLGs can squeeze through pores and penetrate fibrous tissue, bind surface receptors, and enter cells primarily by fusion with their membranes, whereas more rigid NLGs would be taken up primarily by cell endocytosis. These results suggest that liposome elasticity can be optimally tuned to enhance drug delivery in cancer treatment.

**Figure 1:**
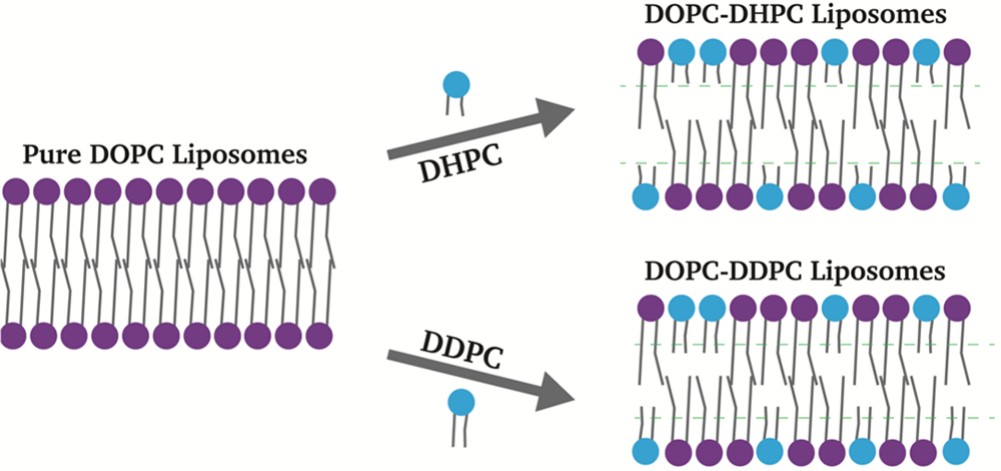
Incorporation of short acyl chain lipids influences lipid packing in the bilayers of DOPC- (or DPMPC-) based liposomes.

In follow-up studies, Large et al.^27,28^ investigated other ways of developing highly elastic liposomes by using formulations primarily composed of the unsaturated phospholipid 1,2-dioleoyl-*sn*-glycero-3-phosphocholine (DOPC) or 1,2-dipalmitoleoyl-*sn*-glycero-3-phosphocholine (termed here DPMPC). These lipids had either 18 or 16 carbon atoms and a double bond linking carbon atoms 9 and 10 in both of their acyl tails. DOPC or DPMPC was mixed with the shorter saturated lipid 1,2-diheptanoyl-*sn*-glycero-3-phosphocholine (DHPC, with 7 carbon atoms in their acyl tails) or 1,2-didecanoyl-*sn*-glycero-3-phosphocholine (DDPC, 10 carbon atoms) in a molar ratio of 75:25 (Table 1). The resulting liposomes had mean diameters ranging between 87 and 92 nm. Their results^27,28^ indicate that the 75:25 liposomes were softer than their pure DOPC or DPMPC counterparts, with stretching moduli between 145 and 166 mN/m, as determined experimentally from micropipette aspiration. These values are 14–33% smaller than those obtained for pure DOPC (216 mN/m) or DPMPC (193 mN/m) liposomes. Pure DOPC or DPMPC liposomes with an aqueous core had elastic (Young’s) moduli ≥45 kPa. Furthermore, the mixed liposomes had lysis tensions (the tension at which the membrane ruptures) that were up to 55% lower than the values observed in pure DOPC or DPMPC liposomes. In *in vitro* experiments, the mixed liposome formulations showed significantly increased internalization by cancer and healthy control cells, increased (2-fold) transendothelial penetration in a cancer-associated vascular endothelium model, and increased (1.9-fold) spheroid penetration, all relative to pure DOPC or DPMPC liposomes. In *in vivo* experiments, the mixed liposome formulations exhibited up to 2-fold-higher tumor accumulation relative to the pure DPMPC liposomes. The incorporation of short acyl chain lipids was postulated to alter the lipid packing in these liposomes (Figure 1). Lipids in pure DOPC or DPMPC liposomes have a “kink” in their unsaturated acyl chains, which arises from the double bond that sterically disturbs packing in the acyl chains. This effect is structurally observed by the 42% increase in the lipid head area of DOPC, relative to its saturated counterpart 1,2-distearoyl-*sn*-glycero-3-phosphocholine (DSPC) in the liquid-crystalline phase.^29,30^ When DHPC or DDPC is added to DOPC or DPMPC liposomes, the saturated sections of the acyl chains of both short and long lipids pack closely up to the double bond (Table 1). This packing effect increases the density of acyl chains per area and relieves steric hindrance after the double bond (Figure 1). Taken together, the combination of different lengths and saturation of lipid acyl chains alters the mechanical properties of the lipid bilayer, yielding nanoparticles with unique deformability.

**Table 1:**
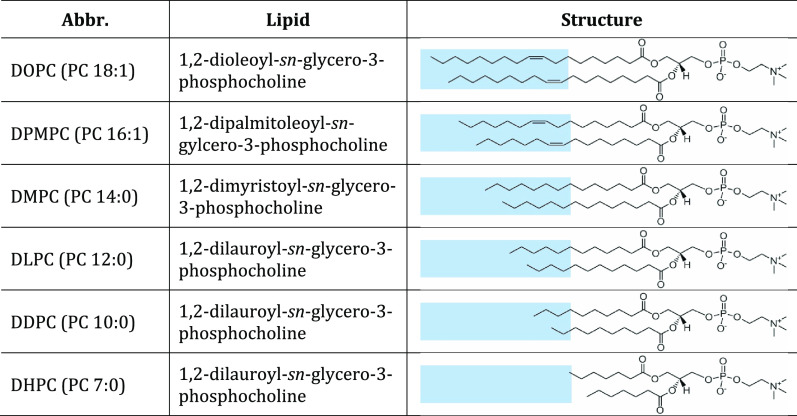
Structures of the Lipids Studied in This Work^a^

a The light-blue shaded area highlights the sections that are different between the lipids. Double bonds in DOPC and DPMPC are between the 9th and 10th carbon atoms in both acyl chains.

Molecular dynamics (MD) simulations, with their “computational microscope” capabilities, are uniquely positioned to provide insights at the atomic level of detail and thus help evaluate the postulates described above. As the experimental liposomes had diameters of ∼90 nm, here we considered just a representative section of them and neglected curvature effects by modeling lipid bilayers with DOPC or DPMPC as the primary component mixed with DHPC or DDPC as the secondary component. We also considered the saturated lipids 1,2-dilauroyl-*sn*-glycero-3-phosphocholine (DLPC, with acyl tails of 12 carbon atoms) and 1,2-dimyristoyl-*sn*-glycero-3-phosphocholine (DMPC, 14 carbon atoms in its tails) to evaluate how secondary, saturated lipids with tails longer than the position of the double bond in DOPC or DPMPC would alter the packing in our lipid bilayers (Table 1). The mole fraction of primary lipid DOPC or DPMPC was varied between 100% and 65%. Most of our MD simulations used all-atom (AA) models, but we also performed simulations with coarse-grained (CG) models to consider lipid bilayers with larger *x*–*y* areas (∼30 × 30 nm^2^ compared to ∼8 × 8 nm^2^ in our atomistic simulations) to assess the possible formation of lipid nanodomains in our systems.

MD simulations have been extensively used to model lipid bilayers and even realistic computational representations of cell membranes.^31^ A number of studies focused on which molecules can soften lipid bilayers and how that happens, with a fraction of them considering DPMPC, DOPC, or DPMPC lipids. In the study by Akhunzada et al.,^32,33^ the properties of a pure DOPC bilayer were compared against those where Rhodamine B (RHB) was attached to some of the lipids. From AA MD simulations, the pure DOPC bilayer had an area per lipid of 68.9 Å^2^ and a thickness of 38.6 Å; the corresponding values for the DOPC-RHB bilayer were 69.2 Å^2^ and 38.2 Å, respectively. These studies also found that RHB affected the lateral diffusion of the lipids. Interestingly, several groups have reported that the bending rigidity of DOPC bilayers does not increase when cholesterol is added,^34−38^ in contrast to what is observed in bilayers of saturated lipids, where cholesterol increases the bending modulus. However, Chakraborty et al.^39^ found that cholesterol increases the bending rigidity of DOPC bilayers through a combination of neutron spin–echo spectroscopy, solid-state deuterium NMR spectroscopy, and atomistic MD simulations, sparking several follow-up reports.^40−43^ Alves et al.^44^ determined forces and energies required for fullerene C_60_ to partition into DOPC bilayers with cholesterol. Their AA MD simulations showed that the presence of cholesterol in the lipid bilayers increases the membrane rigidity, affecting the force needed to insert or extract C_60_. Saeedimasine et al.^45^ used atomistic and CG MD simulations to analyze the structural and mechanical properties of the sphingomyelin or galactosylceramide lipids mixed with phospholipids and cholesterol. Doktorova et al. recently proposed a novel method to estimate the area compressibility modulus from a single simulation,^46^ obtaining values for several lipid bilayers, including DOPC. Likewise, based on simulations with the CG Martini force field, Braun and Sachs^47^ presented a new algorithm to determine the membrane structure, area per lipid, and bending rigidity from MD simulations of lipid vesicles. Wang et al.^48^ used CG MD simulations to construct the phase diagram of DPPC lipid bilayers in the presence of varying cholesterol concentrations and temperatures. Chng et al.^49^ used MD simulations with the Martini force field to investigate the peroxidation of lipids with polyunsaturated fatty acid tails. They found that peroxidation at sites in the bilayer interior disturbs and softens the membrane, whereas peroxidation at sites near the membrane–water interface results in a more ordered and stiffer membrane. To the best of our knowledge, mixed bilayers involving the lipids mentioned in Table 1 have not been studied before through MD simulations. The rest of this article is structured as follows: computational models and methods are described in Section 2, our results are presented and discussed in Section 3, and our concluding remarks are included in Section 4.

## 2 Methodology

### 2.1 System Setup

The different lipids investigated in this work are listed in Table 1. For clarity, the term “primary lipids” refers to DOPC and DPMPC, which have long unsaturated acyl chains and a larger molar fraction in our bilayer systems. “Secondary lipids” refers to DHPC, DDPC, DLPC, and DMPC, which have shorter saturated acyl chains and smaller mole fractions. In this work, we investigated several properties of DOPC- and DPMPC-based bilayers, including the area compressibility modulus (*K*_*A*_), order parameters (*S*_*C*_), lateral diffusion coefficients, and headgroup distributions and how they are affected by the different types and molar ratios of the secondary lipids. Pure lipid bilayers and binary mixed bilayers composed of one primary lipid and one secondary lipid with several molar ratios (95:5, 85:15, 75:25, 65:35, and 90:10 for some systems, see Table S1) were investigated. The initial structures of all our all-atom (AA) bilayer systems were assembled using the heterogeneous lipid generation function in Membrane Builder in CHARMM-GUI.^50,51^ In total, 100 lipids in each leaflet were assembled in a rectangular box, hydrated with 2.25-nm-thick water layers above and below the bilayer. The bilayer area is approximately 8 × 8 nm^2^ in the *x*–*y* plane, with areas per lipid of about 60–65 Å^2^. The ion concentration was set to 150 mM by adding sodium chloride to mimic the normal serum sodium levels. All compositions were equilibrated using the six-step equilibration scheme as suggested by CHARMM-GUI. CHARMM36^52−54^ was used to model lipids and ions, and water molecules were modeled using the TIP3P model.^55^ In these steps, the restraints on lipids were gradually reduced during equilibration simulations over 2.25 ns. We also investigated DOPC:DPPC (75:25), DOPC:DLPC (75:25), and DOPC:DTPC (75:25) bilayers using the CG Martini v3.0^56,57^ force field (here the Martini lipid names and parameters were used as indicated on their website, http://www.cgmartini.nl). Table 2 shows Martini representations of the lipids considered in these CG simulations. The Insane^58^ Python script was used to generate our initial Martini configurations, which had *x*–*y* areas of approximately 30 × 30 nm^2^, with a total of 1520 lipids per leaflet.

**Table 2:**
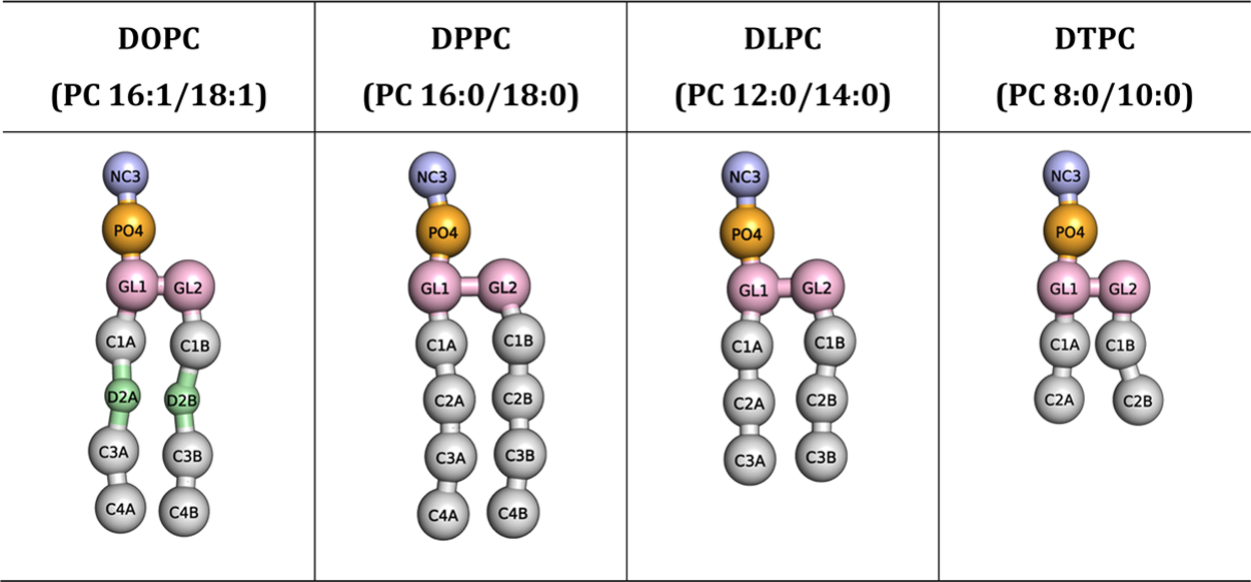
Martini Representations of Lipids^a^ Considered in Our CG Simulations^b^

a See also Table 1.

b Labels represent Martini bead types. The “Martini” lipid names were used.

### 2.2 Simulation Parameters

All AA MD simulations were performed using the NAMD (v3.0-GPU) simulation package,^59^ whereas the GROMACS (v2018.4) simulation package^60−62^ was used for simulations with the Martini force field (FF). In NAMD, a 350 ns production run with a 2 fs time step was performed after the equilibration stages in the *NP*_*zz*_*γT* ensemble (i.e., constant number of molecules *N*, normal pressure *P*_*zz*_, surface tension γ, and temperature *T*). Langevin dynamics and the Nosé–Hoover Langevin Piston algorithm^63,64^ were respectively used to maintain the temperature at *T* = 298 K and control the components of the pressure tensor at the desired values. The normal pressure component was kept fixed at *P*_*zz*_ = 1 bar for all systems. We ran our AA simulations at surface tensions of γ = −7, 0, 7, and 15 mN/m, which are related to the components of the pressure tensor by^65−68^
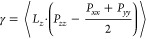
1where *L*_*z*_ is the dimension in the *z* direction of the simulation box and *P*_*xx*_, *P*_*yy*_, and *P*_*zz*_ are the lateral and normal components of the pressure tensor. All bonds involving hydrogen atoms were constrained using the ShakeH algorithm.^69^ The particle mesh Ewald method^70^ with a real-space cutoff of 1.2 nm was used for electrostatic interactions by applying a shifting function to the electrostatic potential at the cutoff distance, and van der Waals interactions were cut off at 1.0 nm and smoothly reduced to zero at 1.2 nm using the force-based switching option.

For our CG simulations with GROMACS, the leapfrog algorithm was used for solving Newton’s equation of motion with an integration time step of 20 fs. The membrane and solvent temperature were fixed at *T* = 298 K separately using the v-rescale thermostat^71^ with a coupling constant of 1 ps. During the equilibration stages, all components of the pressure were fixed at 1 bar (γ = 0 mN/m) by a Berendsen barostat with semi-isotropic pressure coupling, with a time constant of 5 ps and a compressibility of 4.5 × 10^–5^ bar^–1^. For production runs, a Parrinello–Rahman barostat with semi-isotropic pressure coupling was used, with a time constant of 12.0 ps.^72^ The electrostatic and Lennard-Jones interactions were cut off with a real-space cutoff of 1.1 nm, and the dielectric constant (ε_*r*_) was set to 15, the default value used in the Martini force field.^73^ Electrostatics were handled using the reaction field method^74^ with a cutoff value of 1.1 nm and a relative permittivity of ε_*r*_ = 15.

### 2.3 Simulation Analysis

#### 2.3.1 Area per Lipid and Bilayer Thickness

The average area per lipid, also known as the headgroup area, was calculated by dividing the average box lateral area by the total amount of lipids in one leaflet. The bilayer thickness was calculated by subtracting the two average values of the *z* coordinates of the phosphorus atoms in the two leaflets. Because of different surface tensions, the bilayer thickness and surface area change synchronously. In addition, the bilayer thickness is affected by the degree of embedding of the two leaflets.

#### 2.3.2 Area Compressibility Modulus *K*_*A*_

Young’s modulus *E* is a mechanical property used to quantify the elasticity of bilayers and measure liposome stiffness in experiments, which is determined by measuring the tensile deformation:
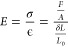
2

Likewise, the area compressibility modulus *K*_*A*_ is the derivative of tensile as a function of area strain, which also serves as a measure of bilayer stiffness^45,75,76^
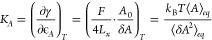
3where γ is the surface tension and ϵ_*A*_ is the area strain defined as
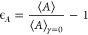
4Calculation of the area compressibility modulus *K*_*A*_ requires the area per lipid results from a bilayer subjected to different surface tensions. Therefore, all atomistic bilayers shown in Table S1 were simulated under −7, 0, 7, and 15 mN/m surface tensions in NAMD by performing *NP*_*zz*_*γT* simulations. Uncertainties in *K*_*A*_ were computed from the standard uncertainties in our area per lipid results, as determined from the autocorrelation method described by Grossfield et al.^77^

#### 2.3.3 Order Parameters

Lipid tail order parameters (*S*_*C*_) are the most common method to account for the ordering of lipids in bilayers. These order parameters can be measured experimentally by the quadrupolar splitting method in the NMR spectra of deuterium nuclei or by the dipolar splitting method in ^13^C NMR, providing information about the overall order of the membrane and specific functional groups in the lipids.^78−81^ Several recent studies have shown good agreement between computation and experiment.^45,76,82−84^*S*_*C*_ is defined as
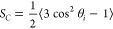
5where θ_*i*_ is the angle between the *z* coordinate and a vector joining carbon atoms *i* – 1 and *i* + 1 in the lipid acyl tails. As the order parameter can exhibit variations along the length of the alkane tail of lipids, it is common to report average values of order parameters as a function of the position of the carbon atoms along the lipid tail.

#### 2.3.4 Lateral Diffusion Coefficients

The movement of lipids inside bilayers can lead to lipid aggregation and the formation of membrane pores and can potentially serve as a measure of the stiffness of bilayer. For example, previous studies have shown that increasing the amount of cholesterol (which usually stiffens lipid bilayers) leads to a reduction in the lateral diffusivities of lipids such as DPPC.^85^ Lateral diffusion coefficients of lipids were determined from the mean squared displacements (MSD) using the following Einstein relation, choosing the phosphorus atoms as the reference points:
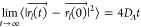
6Both finite size and hydrodynamic effects can influence the lateral diffusion of lipid molecules.^86,87^ Klauda et al.^86^ observed a significant reduction in the lateral diffusion constant in bilayer simulations as the total lipid count increased from 72 to 288. However, they also reported that structural properties such as electron density and deuterium order parameters were unaffected by the studied variations in the system size. As discussed below, agreement with previous experimental and simulation results for area per lipid, bilayer thickness, and area compressibility modulus (Sections 3.1 and 3.2) suggests that finite-size effects did not affect the calculation of these properties in our simulations. In contrast, our diffusivity results for pure DOPC bilayers are higher than the previously reported simulation and experimental results. Therefore, possible finite-size effects in our diffusivity results are discussed in Section 3.5. In addition, Venable et al.^87^ emphasized the significance of hydrodynamic effects when examining lipid and peptide behaviors in biological membranes. Hydrodynamic effects were not considered in our study.

#### 2.3.5 Voronoi Diagrams

Nanodomains could form in mixed lipid bilayers as induced by stress,^88^ the presence of immobilized particles,^89^ and additives such as hydrophobic compounds^90^ and sugars.^91^ These nanodomains can be small or transient and thus might be difficult to detect in experiments. As the limited system size in our atomistic MD simulations could potentially constrain nanodomains, we performed CG MD simulations considering systems with larger *x*–*y* areas (∼30 × 30 nm^2^). Voronoi diagrams were used to examine the lipid distributions from our CG simulation trajectories. Such diagrams are composed of partitions on a plane identified as Voronoi cells, with each cells partitioned on the basis of a seeded center point such that all points within the cell are closer to its seed than to any other.^92^ Voronoi diagrams can be used to calculate local values of the area per lipid in each leaflet. Likewise, Voronoi diagrams were used for measuring and representing the surface area of each lipid molecule in our systems, where the phosphate groups (PO_4_ beads in CG Martini) were used as seeds. The *Freud* library was used to plot and analyze the Voronoi diagrams.^93^

## 3 Results and Discussion

### 3.1 Area per Lipid and Bilayer Thickness

**Figure 2:**
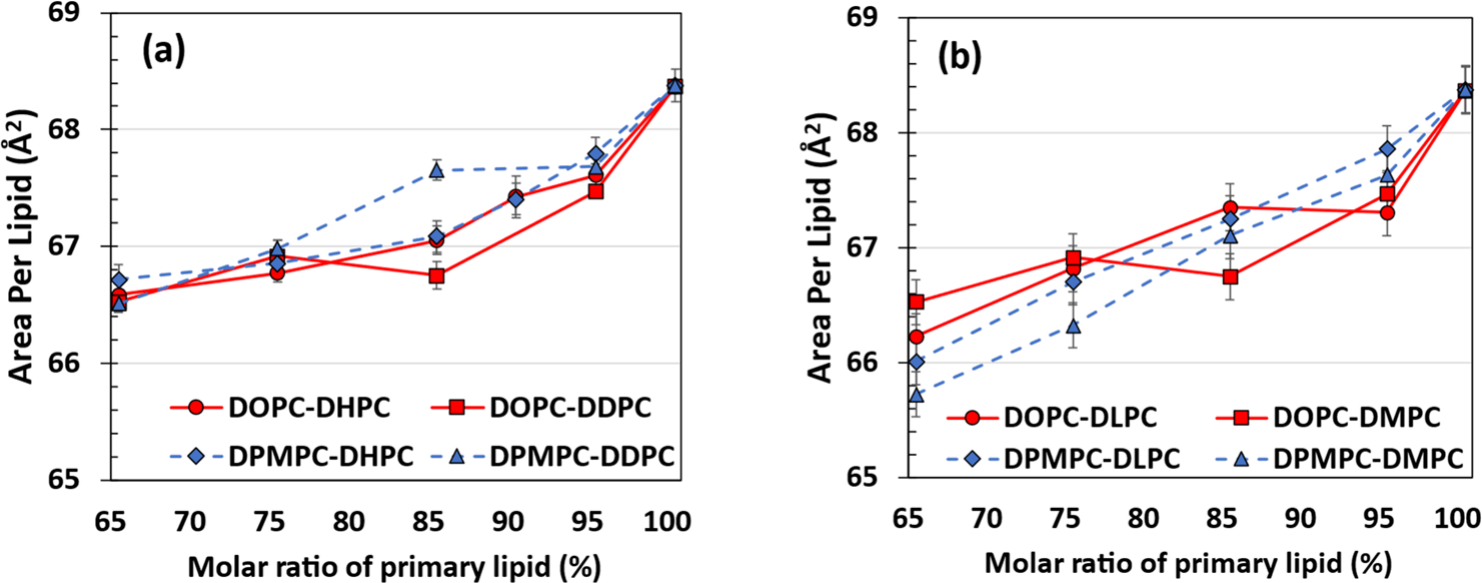
Area per lipid of different bilayers as a function of the mole fraction of DOPC/DPMPC. In both figures, DOPC-based bilayers are shown by red solid lines and DPMPC-based bilayers are shown by blue dashed lines. (a) DOPC-DHPC = red circles; DOPC-DDPC = red squares; DPMPC-DHPC = blue diamonds; DPMPC-DDPC = blue triangles. (b) DOPC-DLPC = red circles; DOPC-DMPC = red squares; DPMPC-DLPC = blue diamonds; and DPMPC-DMPC = blue triangles.

Results for the area per lipid (APL) for our systems at a surface tension of γ = 0 mN/m are depicted in Figure 2(a) for DOPC or DPMPC with DHPC or DDPC (the shortest saturated lipids we considered) and in Figure 2(b) for DOPC or DPMPC with DLPC or DMPC. Similar results at surface tensions of γ = −7, 0, 7, and 15 mN/m are shown in Figure S1 (Supporting Information). These results show that pure DOPC and DPMPC bilayers exhibit nearly identical APL at a surface tension of γ = 0 mN/m (Figure 2). Our APL result for a pure DOPC bilayer, 68.37 ± 0.06 Å^2^, is in good agreement with previously reported simulations (67.1 ± 0.5 Å^2^ and 69.0 ± 1.2 Å^2^)^94−96^ and experimental results (72.4 ± 0.5 Å^2^).^97^ We did not find APL results reported for pure DPMPC lipid bilayers. In general, all of the results shown in Figures 2 and S1 indicate that the area per lipid decreases monotonically as the mole fraction of the primary lipid is reduced. Increases in the values of surface tension (i.e., reducing the values of the lateral components of the pressure, *P*_*xx*_ and *P*_*yy*_, as *P*_*zz*_ is kept fixed at 1 bar, see eqn 1) tend to increase the values of APL for all systems (Figure S1). In general, the four lines in all plots shown in Figures 2 and S1 are relatively close to each other, with the largest differences in APL values being on the order of 1 Å^2^ for some of our systems. These observations suggest that the type of primary or secondary lipid does not have a marked influence on the APL values. This conclusion was somewhat expected, given that the six lipids considered have the same headgroup (Table 1). At any given system composition, variations in the length of the acyl chains of the primary or secondary lipid are not expected to significantly affect the surface area in the *x*–*y* plane of the bilayer. In addition to the area per lipid, bilayer thickness is another relevant metric for analyzing the impact of lipid types and compositions. These results are presented in Figure S2 (Supporting Information) at the four different values of surface tension considered. Our pure DOPC bilayer thickness of 38.36 ± 0.46 Å matches other results from simulations (38.51 ± 0.50 Å)^98^ and experiments (35.30–38.9 Å).^99−103^ Likewise, our pure DPMPC bilayer thickness of 35.1 ± 0.4 Å agrees with the experimental phosphorus–phosphorus distance of around 35 Å, as determined from small-angle X-ray scattering (SAXS) diffraction patterns.^104^ In general, the DOPC-based bilayers are thicker than the DPMPC-based bilayers regardless of the type of secondary lipid, which was expected as DOPC has two additional carbon atoms in its acyl chains. Depending on the secondary lipid, the thickness of bilayers follows the order DHPC < DDPC < DLPC < DMPC (Figure S2).

**Figure 3:**
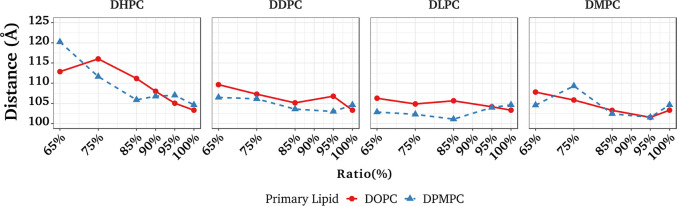
Sum of the standard deviations (distances, in Å) from the average *z* coordinate of phosphorus atoms, as indicators of the surface unevenness of the bilayer structure. These values were calculated over 500 random simulation frames (see the text for details of how these summed distances were computed), as a function of the molar ratio of long tail lipids at a surface tension of γ = 0 mN/m. Secondary lipids considered are indicated by the top labels over each plot. Red solid lines with circles = DOPC; blue dashed lines with triangles = DPMPC.

To gain further insight into the results presented in Figures 2 and S1, at 500 random simulation frames of our production runs, we first determined the average position of the *z* coordinate (normal to the bilayer surface) of the phosphorus atoms in the lipid headgroups in each bilayer leaflet, giving us a total of 1000 average values (2 leaflets and 500 random simulation frames; on each leaflet there are 100 lipids). We then computed the standard deviations from the 1000 average values and added the standard deviations. These summed distance values serve as indicators of the surface unevenness of the bilayer structure, with larger values signaling a broader vertical distribution of phosphorus atoms and, consequently, a more uneven *x*–*y* surface. These results are shown in Figure 3 at a surface tension of γ = 0 mN/m and in Figure S3 for all surface tensions considered here. In Figure 3, bilayers comprising the shortest secondary lipid, DHPC, exhibit standard deviation propagations that are 10–20 Å larger than the values observed in systems containing the longest secondary lipid considered, DMPC. This observation suggests that secondary lipids with shorter acyl chains tend to increase the unevenness of lipid bilayers. Larger values of surface tensions resulting from reductions in the lateral pressure components *P*_*xx*_ and *P*_*yy*_ (eq 1) cause the bilayers to slightly expand in the *x* and *y* directions, resulting in a narrower distribution of phosphorus atoms and headgroups as we go down the rows in Figure S3. In addition, the results shown in Figures 3 and S3 indicate that all bilayer surfaces become increasingly uneven as the molar ratio of the primary lipids decreases. This observation suggests that misaligned headgroups in uneven bilayers create more room for the bilayers to slightly shrink in the *x*–*y* plane, resulting in smaller areas per lipid in the systems with smaller mole fractions of the long unsaturated lipids (Figure 2).

### 3.2 Area Compressibility Modulus

**Figure 4:**
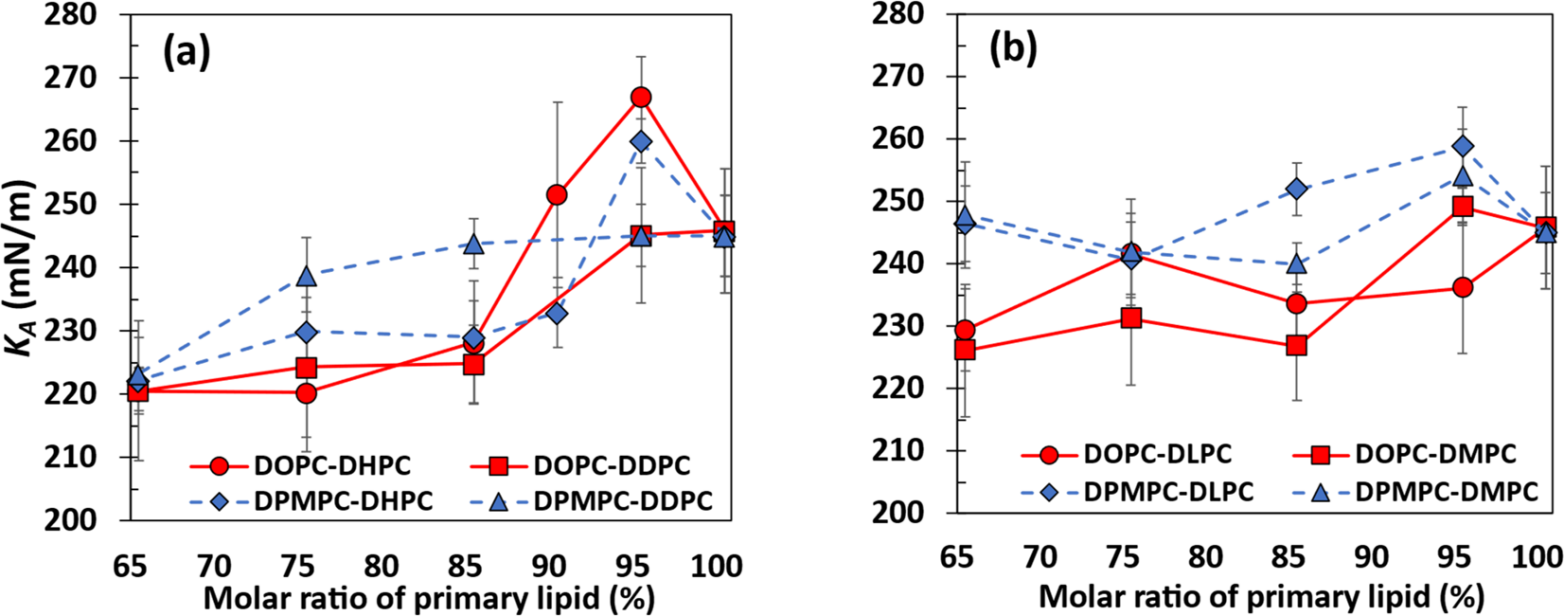
Area compressibility modulus of bilayers with DOPC (red lines) or DPMPC (blue lines) as primary lipids as a function of their molar ratio. (a) DOPC-DHPC = red circles; DOPC-DDPC = red squares; DPMPC-DHPC = blue diamonds; and DPMPC-DDPC = blue triangles. (b) DOPC-DLPC = red circles; DOPC-DMPC = red squares; DPMPC-DLPC = blue diamonds; and DPMPC-DMPC = blue triangles. Data shown in these figures are presented in Table S2.

Figure 4 shows results for the area compressibility modulus *K*_*A*_, where a smaller value indicates a softer lipid bilayer; numerical values are reported in Table S2 (Supporting Information). For a pure DOPC bilayer, we obtained *K*_*A*_ = (245.8 ± 9.9) mN/m; this value is in general agreement with reported experimental (265 ± 18 mN/m and 310 ± 20 mN/m)^105,106^ and computational results (256 ± 17 mN/m, 253 ± 42 mN/m and 246 ± 20 mN/m).^46^ We observe that our computational results align with the lower range of reported experimental and simulation values. Discrepancies might stem from the use of different force fields as well as the inherent limitations of each force field in simulations, fluctuations in pressure during simulations at constant surface tension, differences in barostats and parameters in simulations, and the use of different methodologies for the calculation of *K*_*A*_ in simulations. For a pure DPMPC bilayer, we obtained *K*_*A*_ = (245.0 ± 6.4) mN/m. Although we could not find *K*_*A*_ results for DPMPC in the literature, the fact that both pure DOPC and DPMPC bilayers had statistically similar values of *K*_*A*_ is in qualitative agreement with the experimental observation that pure DOPC and DPMPC liposomes had statistically similar values of stretching moduli, as determined from micropipette aspiration measurements.^27,28^ For most compositions, DOPC and DPMPC bilayers mixed with DHPC or DDPC (Figure 4a) tend to have smaller values of *K*_*A*_ compared with pure bilayers. The exceptions to this observation seem to be the DOPC-DHPC (95:5) and DPMPC-DHPC (90:10, 95:5) bilayers, which seem to have values of *K*_*A*_ larger than those of the pure bilayer systems. In general, *K*_*A*_ decreases as the molar ratio of the primary lipid is reduced for bilayers composed with DHPC or DDPC, although for most systems reductions in the mole fraction of the primary lipid beyond 85% do not seem to lead to statistically significant drops in *K*_*A*_. The only exception is the DPMPC:DDPC (65:35) system, which has the smallest *K*_*A*_ among the composition ranges examined for this particular mixed bilayer. Our results indicate that adding 25–35% of the secondary lipid DHPC or DDPC to DOPC- or DPMPC-based bilayers can reduce *K*_*A*_ by up to 10.4% compared to the values observed in pure DOPC or DPMPC lipid bilayers. As the uncertainties in the data reported in Figure 4 and Table S2 are relatively large, a two-sample *t* test was performed to assess whether the *K*_*A*_ values of all mixed bilayers considered are statistically different from the value observed in the equivalent pure bilayer system with the same primary lipid. The *t* test was conducted at a 95% confidence level with degrees of freedom computed as *df* = *n*_1_*+ n*_2_ – 2. As all *K*_*A*_ values were determined from simulations at four values of surface tension, *n*_1_ = *n*_2_ = 4, giving *df* = 6. From the table of critical values of *t* for two-tailed tests,^107^ we obtain *t* = 2.447. Therefore, systems with absolute values of *t* larger than 2.447 (marked in red in Table S2, Supporting Information) indicate mixed bilayers that have *K*_*A*_ values that are statistically different from the values observed in pure bilayers. From our *t* test, *p* values were also determined and are reported in Table S2. Likewise, systems with *p* values smaller than 0.05 (also marked in red in Table S2) denote those mixed bilayers with *K*_*A*_ values that are statistically different from the results for pure systems. In general, these results indicate that all mixed systems with 35% DHPC or DDPC as the secondary lipid have *K*_*A*_ values that are smaller and statistically different from the values observed in pure DOPC or DPMPC systems. The same observation applies to all systems having 15% and 25% DHPC and to the mixed DOPC systems with 15% and 25% DDPC.

Our observations of the stiffness of the pure DOPC and DPMPC systems, compared to the mixed bilayers DOPC:DHPC (75:25), DPMPC:DHPC (75:25), DOPC:DDPC (75:25), and DPMPC:DDPC (75:25), are in agreement with the trends observed in our experimental results for the stretching modulus of liposomes with the same compositions.^27,28^ The experimental stretching modulus values of DOPC:DHPC (75:25) and DPMPC:DHPC (75:25) liposomes were 155 and 165 mN/m, both smaller than the values determined for pure DOPC (218 mN/m) and pure DPMPC (196 mN/m) liposomes. In our simulations (Figure 4a and Table S2), *K*_*A*_ of the DOPC:DHPC (75:25) bilayer is 220.3 mN/m, smaller than the values obtained for its DPMPC:DHPC counterpart (229.9 mN/m) and for pure DOPC (245.8 mN/m) and DPMPC (245.0 mN/m). For liposomes with DDPC, the experimental results^27,28^ show a slightly smaller stretching modulus for DOPC:DDPC (75:25), 145 mN/m, compared to that for DPMPC:DDPC (75:25), 151 mN/m, both of which are in turn smaller than the values measured in pure DOPC and DPMPC liposomes. Our simulation results agree well with these observations, as the DOPC:DDPC (75:25) system has *K*_*A*_ = 224.3 mN/m, smaller than the value determined for its DPMPC:DDPC counterpart (*K*_*A*_ = 238.9 mN/m).

For bilayers containing DLPC or DMPC, Figure 4b illustrates that the DPMPC-based bilayers generally exhibit larger *K*_*A*_ (or similar values for some concentrations) compared to that for DOPC-based bilayers, by analogy to what was observed for the DOPC-DDPC and DPMPC-DDPC bilayers (Figure 4a). However, for any given composition, adding DLPC or DMPC results in smaller reductions in *K*_*A*_ with respect to the values of pure DOPC or DPMPC bilayers, compared to what is observed when similar mole fractions of DHPC or DDPC are considered. For example, pure DPMPC bilayers and their binary mixtures with DLPC or DMPC have statistically similar values of *K*_*A*_, and for some concentrations, *K*_*A*_ even increases (Figure 4b and Table S2). Similarly, adding DLPC or DMPC to DOPC-based bilayers led to slightly smaller drops in *K*_*A*_, compared to when DHPC or DDPC is added The most significant drop in *K*_*A*_ relative to the pure bilayer systems observed in the systems depicted in Figure 4b was 8.02% for the DOPC-DMPC (65:35) system, slightly smaller than the maximum drop of 10.4% observed for the DOPC-DHPC (75:25) bilayer (Figure 4a).

In summary, Figure 4 and Table S2 demonstrate that adding significant amounts (i.e., 15 mol % or larger) of any of the four secondary lipids analyzed can make DOPC- or DPMPC-based bilayers less stiff. The largest reductions in *K*_*A*_ values (approximately 10.4%) compared to pure bilayer systems occur when systems consist of 25–35% of the shortest lipids, DHPC (7 carbon atoms in both acyl tails) or DDPC (10 carbon atoms). Conversely, incorporating DLPC (12 carbon atoms) or DMPC (14 carbon atoms) leads, in general, to slightly smaller drops in *K*_*A*_ values (a maximum of 8.0%). DLPC and DMPC have tails that are more comparable in size to those of DOPC (18 carbon atoms) and DPMPC (16 carbon atoms), which have their double bond between the 9th and 10th carbon atoms in both acyl tails (Table 1). Conversely, some of the mixed systems (e.g., some of the 95:5 systems shown in Figure 4) have *K*_*A*_ values that are up to 8.6% larger than the area compressibility moduli of pure DOPC or DPMPC systems. After analyzing additional properties of these systems (see the sections below), we could not offer insights on the possible reasons behind these unexpected increases in *K*_*A*_. One alternative approach, which we did not attempt here, is to compare area compressibility moduli and other properties such as bending moduli in these bilayer systems, as determined from different methodologies^108−111^ that might have smaller uncertainties compared to the calculations reported here.

### 3.3 Order Parameters

**Figure 5:**
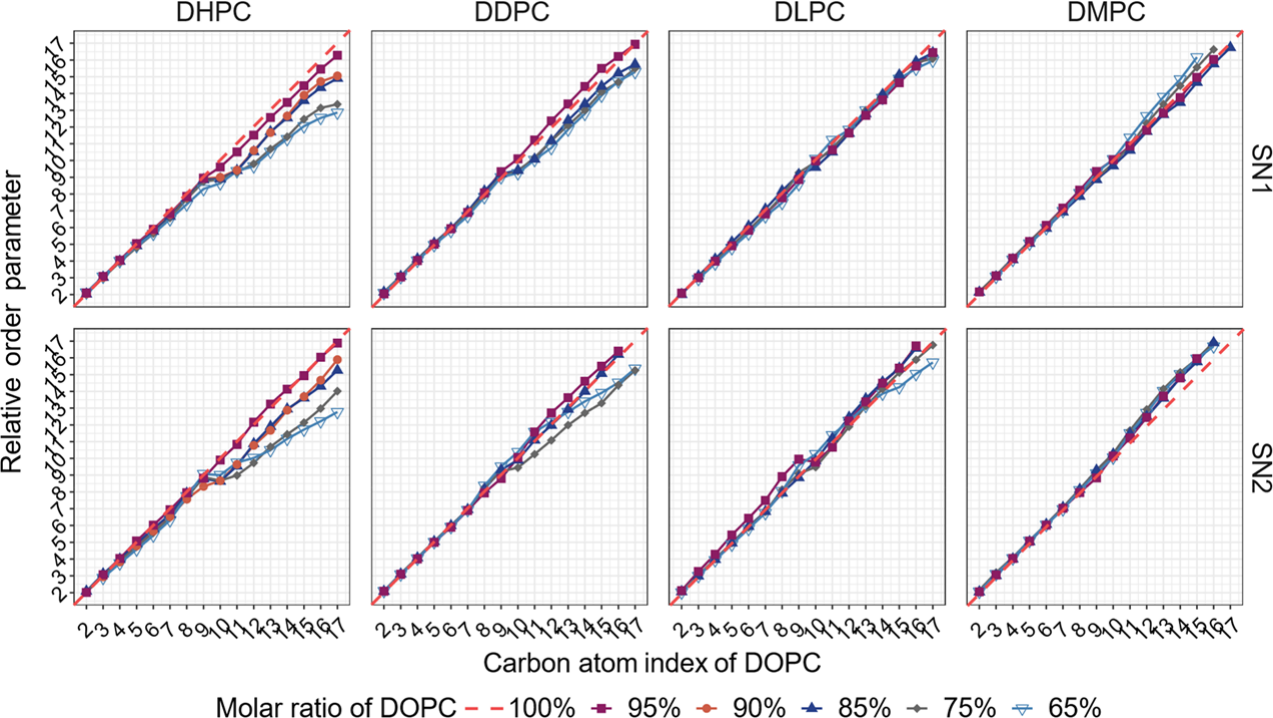
Relative order parameters of two acyl chains SN1 and SN2 for the primary lipid in DOPC-based bilayers. Relative order parameters are determined by dividing the value of the order parameter of each carbon atom in each tail SN1 and SN2 in a mixed bilayer by its counterpart in a pure bilayer system composed of the same primary lipid at the same value of surface tension (γ = 0 mN/m) and then multiplying by the carbon atom index. Therefore, the diagonal line corresponds to the pure DOPC system.

**Figure 6:**
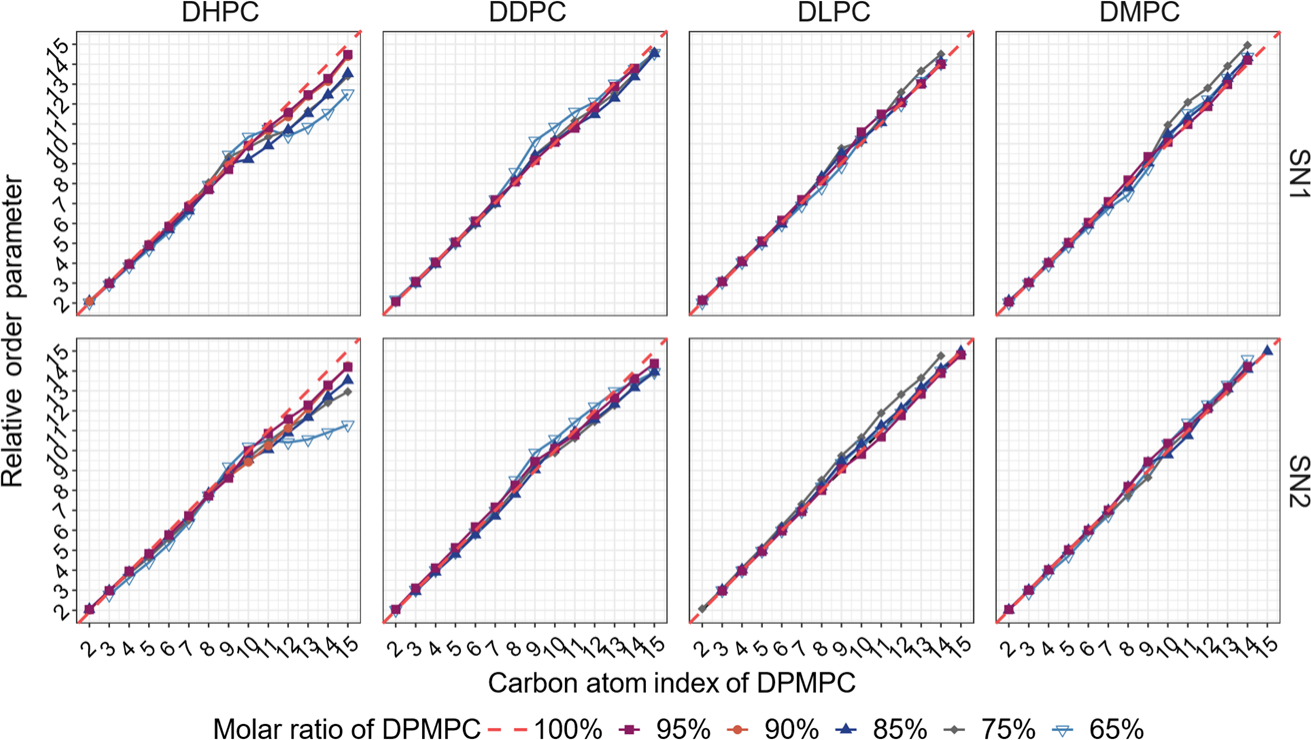
Relative order parameters of two acyl chains SN1 and SN2 for the primary lipid in DPMPC-based bilayers. Relative order parameters are determined by dividing the values of the order parameter of each carbon atom in each tail SN1 and SN2 in a mixed bilayer by their counterparts in a pure bilayer system composed of the same primary lipid at the same value of surface tension (γ = 0 mN/m) and then multiplying by the carbon atom index. Therefore, the diagonal line corresponds to the pure DPMPC system.

To further understand how the type and composition of secondary lipids affect ordering in the acyl tails of the lipids, we measured the order parameters *S*_*C*_ of the lipid acyl tails for our systems with surface tension γ = 0 mN/m. Variations in order parameters could be linked to changes in the stiffness of the lipid bilayers. For example, lipids with high values of order parameters tend to pack more tightly in bilayers, making them more resistant to area changes upon variations in lateral pressure (i.e., variations in surface tension), resulting in larger values of the area compressibility modulus.^39,112^ In addition, the presence of double bonds in lipids such as DOPC or DPMPC introduces kinks in the acyl chains, which results in less ordering of the acyl chains compared with bilayers formed by saturated lipids of similar chain length. As a result, the average order parameters of bilayers formed by unsaturated lipids are generally lower than the values observed for bilayers of saturated lipids of the same chain length. Each primary or secondary lipid in our bilayers is composed of two acyl chains with an equal number of carbon atoms. The order parameters of the primary lipids are depicted in Figures S4 (DOPC-based systems) and S5 (DPMPC-based systems) in the Supporting Information file, where the rows represent results for the two acyl tails, the columns represent the secondary lipids considered, and the different lines in each plot represent the mole fractions studied. The same information is presented in a different way in Figure S6 (Supporting Information), where now the columns represent fixed composition values, and the different lines in each plot correspond to the four secondary lipids considered. Similarly, the same results are presented in terms of relative order parameters in Figures 5 (DOPC-based systems) and 6 (DPMPC-based systems). These relative order parameters were calculated by dividing the value of the order parameter of each carbon atom in each tail SN1 and SN2 in a mixed bilayer by its counterpart in a pure bilayer system composed of the same primary lipid at the same value of surface tension (γ = 0 mN/m). The resulting value was then multiplied by the index of the carbon atom to obtain the relative order parameters. Therefore, the order parameters of the primary lipids in pure bilayers are represented by the diagonal lines in Figures 5 and 6. These relative order parameter figures provide a useful visualization of the impact of the molar ratio and type of secondary lipid on the order parameters of the primary lipid in our bilayers.

The two DHPC subfigures in Figure 5 show a substantial decrease in the order parameters of both acyl chains of DOPC, as the molar ratio of this primary lipid decreases. In comparison to DHPC systems, the order parameters of DOPC in bilayers with the longer saturated lipid DDPC have smaller reductions as the mole fraction of secondary lipid increases. In contrast, systems having DLPC and DMPC as secondary lipids have much smaller variations in the order parameters of DOPC with respect to the values observed in pure bilayers, and in some systems, even small increases in the order parameters are observed. These observations suggest that DHPC, the shortest saturated lipid considered here with only 7 carbon atoms in its acyl chains, can randomize the spatial distributions of the acyl tails of DOPC more than when DDPC, DLPC, or DMPC is added. This observation supports the postulate depicted in Figure 1, namely, that increases in the molar fraction of DHPC (or DDPC) lead to larger cavity volumes, which in turn allows more disorder in the DOPC tails. Similar observations are also noted in the results shown in Figure 6 for systems where the primary lipid is DPMPC. However, in general, the variations in order parameter are smaller than those observed in similar systems with DOPC, as the former unsaturated lipid has slightly shorter acyl tails (16 carbon atoms vs 18). Comparing DHPC columns (SN1 vs SN2 tails) in Figures 5 and 6, in general the first points that deviate significantly from the diagonal correspond to carbon atom 10, suggesting that the double bond introduces kinks in the tails and makes further carbon atoms have more random spatial distributions (Figure 1). From Figure S6 (Supporting Information), we confirm again that adding a larger amount of secondary lipid (i.e., going to the right in Figure S6) with shorter acyl chains leads to sharper reductions in the order parameters of the primary lipid, compared to when we add the same amount of secondary lipids with longer tails.

In Figure S7 (Supporting Information), we explored the impact of different types and compositions of secondary lipids on their order parameters for systems with surface tension γ = 0 mN/m. The first two rows show the order parameters of SN1 chains, whereas the last two rows are for SN2 chains of the four different secondary lipids. These results reveal that values of order parameters of secondary lipids in general follow the descending order of DMPC > DLPC > DDPC > DHPC. These findings suggest that the spatial distributions of the tails in secondary lipids with shorter acyl chains tend to be more random, compared to systems of the same composition where the secondary lipids have longer acyl chains. No apparent trends in the order parameters of the secondary lipids are identified as the system composition varies. However, we note that the small number of secondary lipid molecules in the simulation, particularly when the primary lipid content is 95%, may have affected the accuracy of the results shown in Figure S7.

In summary, the order parameters of acyl chains in a bilayer are influenced by the types and ratios of the lipids present. The presence of short-chain saturated secondary lipids increases the randomness in the spatial distributions of the acyl tails of the primary unsaturated lipids DOPC and DPMPC, with more pronounced effects observed when the secondary lipids have shorter acyl chains. The acyl tails of DOPC and DPMPC in bilayers with the largest concentrations of DHPC and DDPC exhibit the highest levels of disorder among the systems studied. In turn, these systems with 35% DHPC or DDPC had the smallest values of the area compressibility modulus, suggesting a link between order in the carbon atoms of the acyl tails of the primary lipids and bilayer stiffness.

### 3.4 Vertical Distribution of Terminal Methyl Groups

**Figure 7:**
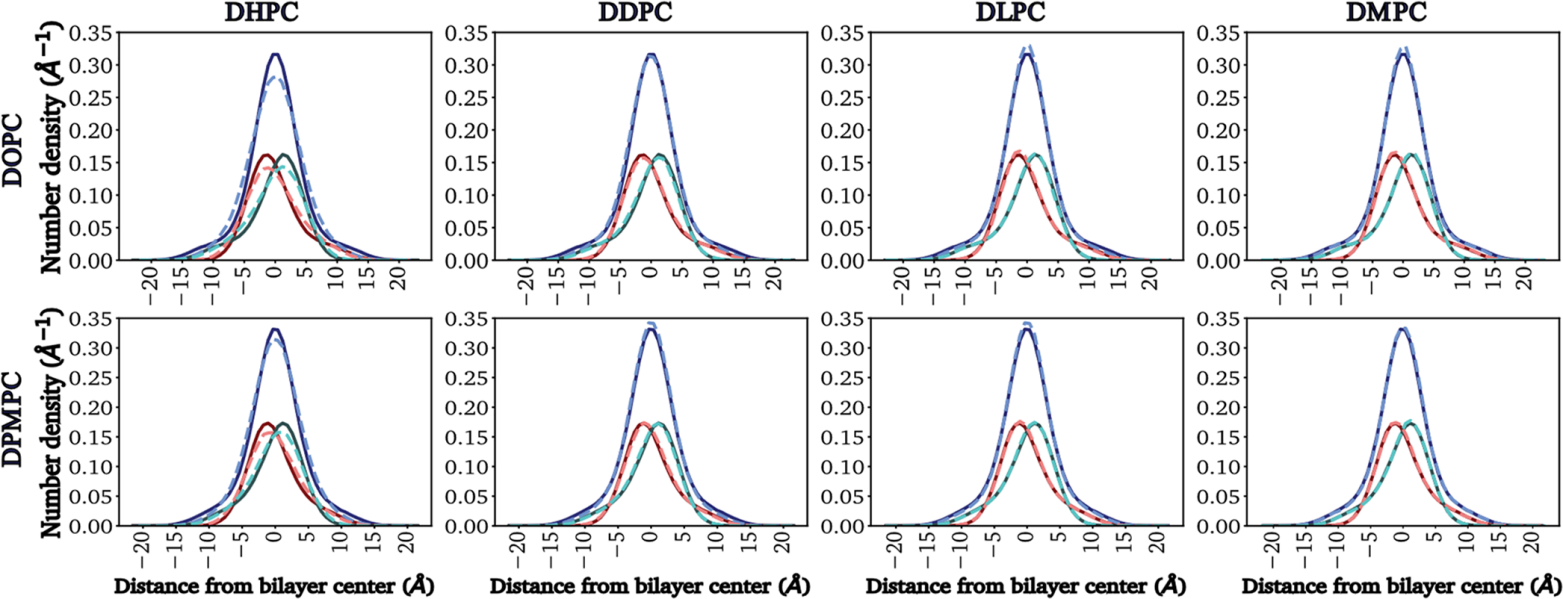
Vertical spatial distribution of terminal methyl groups (TMGs) in primary lipids for systems with 75% DOPC (top row) or DPMPC (bottom row) at a surface tension of γ = 0 mN/m. Results observed in systems with different secondary lipids are shown in vertical columns. Results for mixed bilayers (dashed, light-colored lines) are compared against those for pure bilayers (solid, dark-colored lines). Dark/light red, dark/light green, and dark/light blue represent the TMG distributions for leaflet 1, leaflet 2, and both leaflets, respectively.

The distribution of vertical spatial coordinates of terminal methyl groups (TMGs) of the primary lipids can provide valuable insights into the spatial arrangement of acyl chains within the bilayer. Mihailescu et al.^113^ reported that the *z* coordinates of the lipids’ TMGs in bilayers of DOPC:cholesterol with a molar ratio 2:1 had significantly narrower spatial distributions compared to what is observed in a pure DOPC bilayer. Inspired by that study, here we analyzed the distribution of the TMGs of DOPC or DPMPC in mixed bilayers and compared these results against observations for systems of the same pure primary lipids. These results are shown in Figure 7 for systems with 75% primary lipids DOPC or DPMPC and all secondary lipids studied, in Figure S8 (Supporting Information) for DOPC-DHPC and DPMPC-DHPC systems over the different concentrations examined, all at a surface tension of γ = 0 mN/m, and in Figure S9 (Supporting Information) for systems with 65% primary lipids DOPC or DPMPC and DHPC lipids over four different surface tensions. Solid, dark-colored lines correspond to results in pure DOPC or DPMPC bilayers, while dashed, light-colored lines represent results obtained for mixed bilayers. In all figures, the peaks of the dark- and light-red lines (distribution of TMGs in lipid leaflet 1) are located on the left side of the origin (i.e., below the bilayer center) and the tails expand into positive values of the *z* coordinate, whereas the dark- and light-green lines (distribution of TMGs in leaflet 2) exhibit equivalent behavior (i.e., peaks above the bilayer center and tails expanding well below the bilayer center).

The results shown in Figure 7 indicate that the addition of DMPC, DLPC, and DDPC as secondary components has little effect on the TMG distributions in both DOPC- and DPMPC-based bilayers as compared to results observed in the corresponding pure lipid systems. However, adding increasing concentrations of DHPC (Figures 7, S8, and S9) causes the spatial distributions of TMGs in the primary lipids to have peaks of smaller height and become slightly wider, compared to the results observed in pure bilayers. Interestingly, adding DHPC does not cause the distribution of TMGs to have longer tails; note that on both sides of the distribution peaks, at short distances, the distributions for mixed systems are slightly wider than those of pure systems, but as we travel farther away from the peaks, in general the pure bilayers have longer-tailed distributions compared to the bilayers mixed with DHPC. Furthermore, at any given concentration the distribution of TMGs in DOPC:DHPC systems has smaller peaks that are slightly wider compared to those of DPMPC:DHPC bilayers (Figures 7, S8, and S9), but again the pure lipid systems have distributions with longer tails compared to the mixed DHPC bilayers. The representative simulation snapshots presented in Figure S10 highlight that the distribution of TMGs in primary and secondary lipids in the two leaflets is asymmetric. Interestingly, some of the TMGs venture close to the headgroup regions, as previously observed^113^ in pure DOPC bilayers. Variations in surface tension do not seem to lead to significant changes in the vertical spatial distribution of TMGs in DOPC or DPMPC, as shown in Figure S9 (Supporting Information) for the 65:35 systems with DHPC.

The relationships among the distribution of TMGs, order parameters, and area compressibility modulus suggest that the presence of short-chain saturated secondary lipids, such as DHPC, can increase the disorder in the bilayer’s spatial structure. This remark is supported by the observation of wider TMG distributions, reduced order parameters, and a smaller area compressibility modulus, compared to what is observed in pure bilayer systems. Similar features are also observed in systems with DDPC. In contrast, secondary lipids with longer chains, such as DMPC and DLPC, have minimal impact on the TMG distribution, and the order parameters are similar to those observed in pure bilayer systems; thus, the area compressibility moduli are statistically similar to the values observed in pure DOPC or DPMPC systems.

### 3.5 Lateral Diffusion Coefficients of Lipids

**Figure 8:**
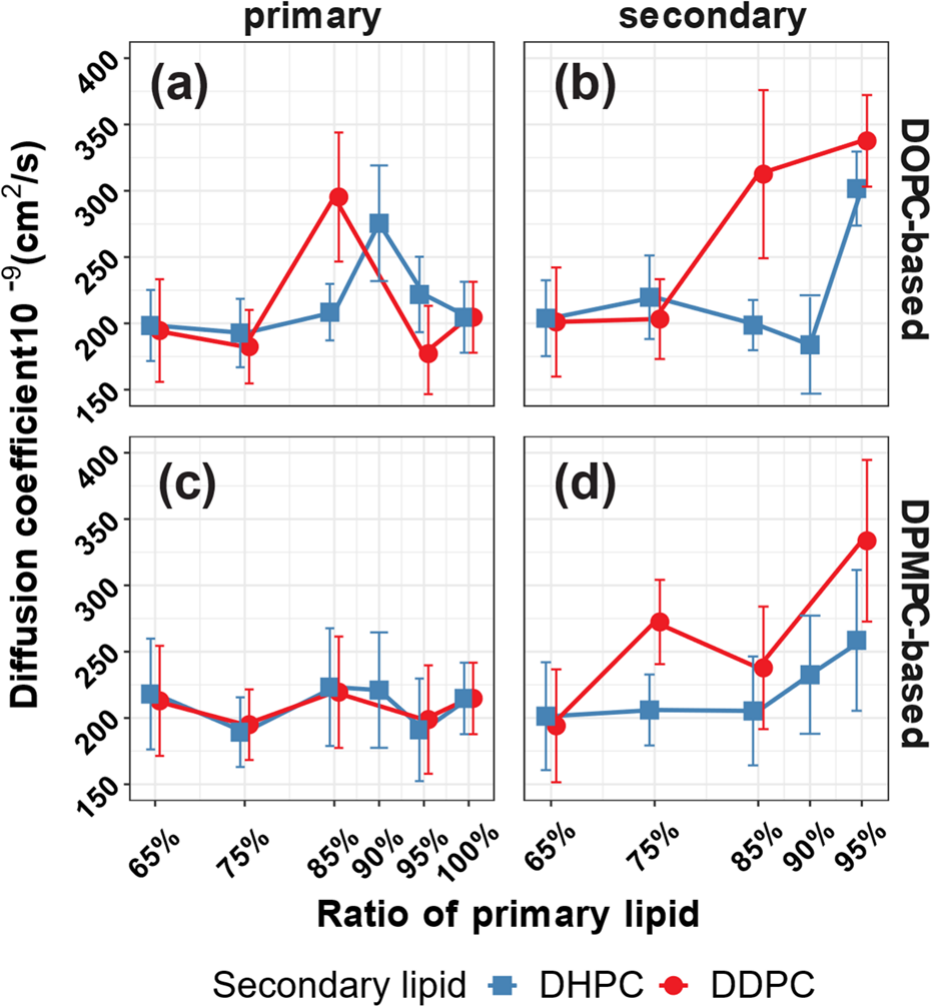
Lateral diffusion coefficients of lipids in bilayers as a function of mole % of the primary lipid, at a surface tension of 0 mN/m. (a) DOPC in DOPC:DHPC (blue) or in DOPC:DDPC (red); (b) DHPC in DOPC:DHPC (blue) or DDPC in DOPC:DDPC (red); (c) DPMPC in DPMPC:DHPC (blue) or in DPMPC:DDPC (red); and (d) DHPC in DPMPC:DHPC (blue) or DDPC in DPMPC:DDPC (red). Although all of the binary mixtures depicted had the same compositions (65, 75, 85, 95, and 100 mol % of the long unsaturated lipid and for some systems 90%), all data points shown were slightly displaced horizontally around these compositions for ease of visualization.

Lateral diffusivities of lipids might be linked to bilayer stiffness, as softer systems are intuitively associated with more fluid, liquidlike bilayers where the lipids have high diffusivities. Conversely, smaller lipid diffusivities may be associated with systems that are less fluid and thus exhibit increased stiffness. Figure 8 shows results for the lateral diffusion coefficients of the lipids in bilayers where DDPC and DHPC are secondary lipids at a surface tension of 0 mN/m; corresponding results for all examined systems at various surface tensions are shown in Figure S11 (Supporting Information). The lateral self-diffusion coefficients of the lipids were calculated from their mean squared displacements (MSDs) by using python scripts developed by Bullerjahn et al.^114^ The Kolmogorov–Smirnov test^114,115^ was applied for detecting possible anomalous diffusion (i.e., those where the MSD does not follow eq 6). The accuracy of the results increases by accounting for correlations between MSD values at different time intervals, as the approach of Bullerjahn et al.^114^ provides an optimal balance between systematic errors (caused by short-time nondiffusive dynamics) and long-time statistical errors (resulting from increasing uncertainties). For pure DOPC bilayers, we obtained ∼200 × 10^–9^ cm^2^/s for the lateral diffusivity. This value is significantly higher than recently reported simulation results (84 × 10^–9^ cm^2^/s),^33^ which in turn agree well with previous simulation and experimental values that range between 50 × 10^–9^ and 140 × 10^–9^ cm^2^/s.^116^ Our simulations did not account for possible hydrodynamic effects, which might affect computed diffusivities.^87^ It is also possible that our all-atom simulations (200 lipids in total) are not large enough and finite-size effects might be affecting our diffusivity results, by analogy to findings reported by Klauda et al. for systems with 72 and 288 lipids.^86^ However, they also reported that structural properties such as electron density and deuterium order parameters were unaffected by the studied variations in system size. As discussed in Sections 3.1 and 3.2, agreement with previous experimental and simulation results for area per lipid, bilayer thickness, and area compressibility modulus suggests that finite-size effects did not affect the calculation of these properties in our simulations. In any case, the diffusivities reported in Figures 8 and S11 need to be viewed with caution and will be discussed only in a qualitative manner below.

The results shown on the left column of Figure 8 for the primary lipids indicate that for most concentrations the red and blue curves are statistically close to each other, suggesting that the lateral diffusivities of the primary lipids DOPC (top left) and DPMPC (bottom left) are similar, regardless of whether they are mixed with DHPC or DDPC. Variations in concentration appear to have minimal impact on the lateral diffusivities of DOPC and DPMPC. Notable exceptions to these observations are the 85:15 DOPC:DDPC system and the 90:10 DOPC:DHPC system (Figure 8a), in which the lateral diffusivity of DOPC sharply increases compared to the values observed at slightly larger or smaller concentrations. The data shown in the right column of Figure 8 demonstrate that in general the lateral diffusivities of the secondary lipids DHPC and DDPC are statistically similar for most concentrations (except the 85% DOPC systems, Figure 8b, and the 75% DPMPC systems, Figure 8d). Furthermore, the lateral diffusivities of the secondary lipids (Figure 8b,d) are in general comparable to the diffusivities of the longer primary lipids (Figure 8a,c). However, the diffusivities of the secondary lipids tend to increase as the concentration of the primary lipid increases beyond 75% (DOPC:DDPC), 85% (DPMPC:DHPC and DPMPC:DDPC), or 90% (DOPC:DHPC), after which the secondary lipids have larger lateral mobilities compared to the primary lipids. In all cases, the lateral diffusion coefficients of the secondary lipid reach their highest value for the systems that have 95% of the primary lipid. However, this observation may be impacted by the small number of molecules of the secondary lipids in these systems, which increases the uncertainty in our measurements. All of these overall observations also apply to systems with the secondary lipids DLPC and DMPC at a surface tension of 0 mN/m (Figure S11). However, the lateral diffusivities of the secondary lipids seem to be significantly affected by variations in the surface tension (Figure S11) without any recognizable trend. In contrast, variations in surface tension do not seem to affect the lateral diffusion coefficients of the primary lipids DOPC and DPMPC. However, the error bars in the diffusivities are the smallest when γ = 0 mN/m (Figure S11), suggesting that changes in surface tension (which in our systems are caused by changes in the lateral pressure) lead to larger variations in the mobilities of the lipids. The observations from Figures 8 and S11 suggest that lipid diffusivities do not correlate with the trends observed in the area compressibility modulus results (Figure 4). The 65:35 systems with DOPC or DPMPC with DHPC or DDPC had the smallest values of *K*_*A*_ (Figure 4), indicating that these systems are softer than pure DOPC or DPMPC bilayers. However, the lateral diffusivities of both primary and secondary lipids remain statistically comparable to the values observed in pure DOPC or DPMPC systems (Figures 8 and S11).

### 3.6 Coarse-Grained (CG) Simulations: Voronoi Diagrams of Lipid Bilayers

**Figure 9:**
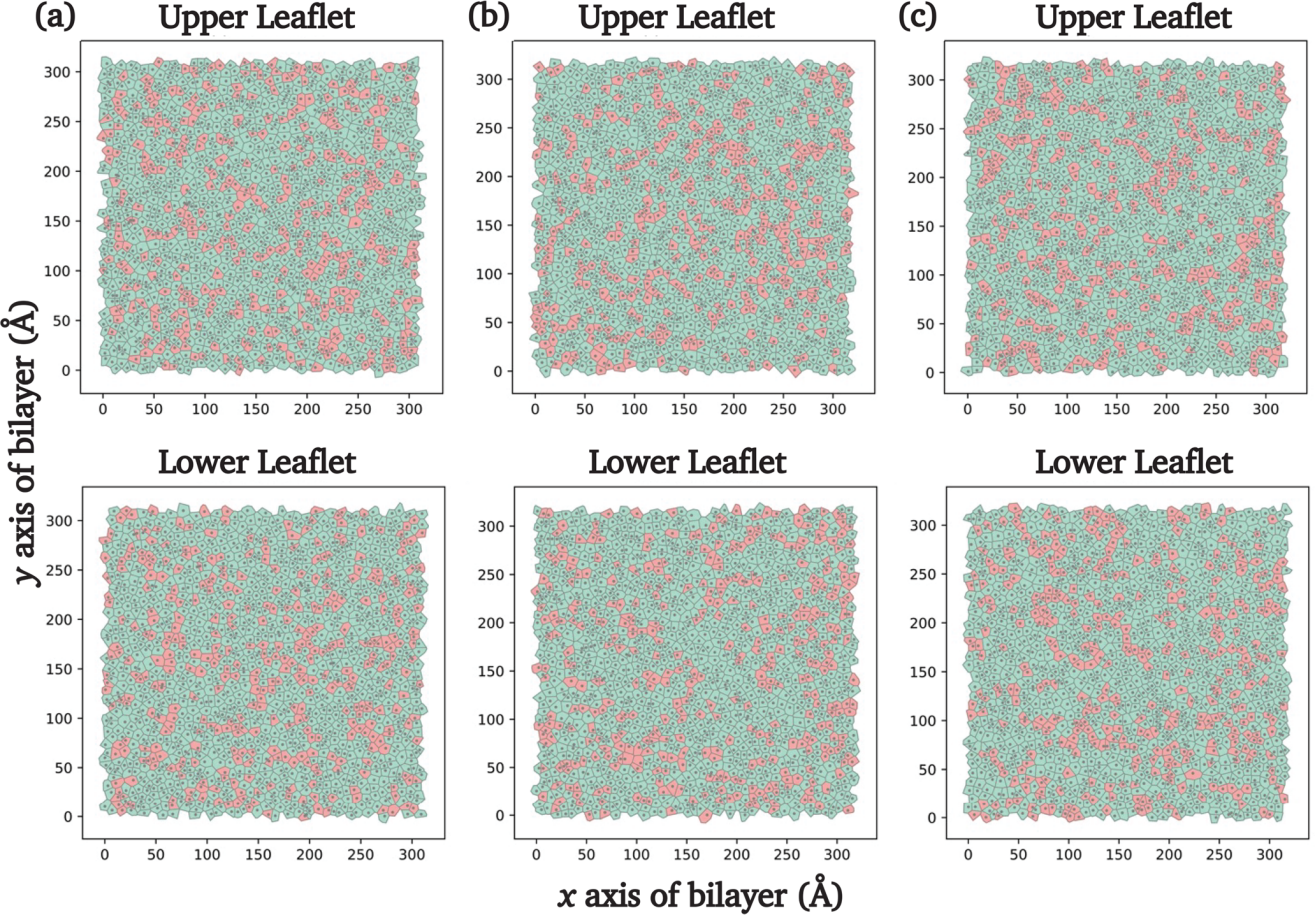
Representative Voronoi diagrams for the 75 mol % DOPC (shown in green) and 25 mol % (a) DPPC (PC 16:0/18:0), (b) DLPC (PC 12:0/14:0), and (c) DTPC (PC 8:0/10:0) membrane bilayers in the Martini force field using GROMACS. Secondary lipids are shown in pink.

The AA simulations provided links between stiffness in our systems, as measured by the area compressibility modulus, and molecular-level properties measuring disorder in the bilayers, specifically, the area per lipid, lipid acyl chain order parameters, and vertical distribution of terminal methyl groups. As bilayers of area ∼8 × 8 nm^2^ for ∼350 ns were studied in these AA simulations, we then ran CG simulations of similar lipid bilayers using the Martini force field and studied systems of area ∼30 × 30 nm^2^ for up to 8 μs to evaluate the possible formation of nanodomains in our systems over these length and time scales. As mentioned earlier, we used four CG lipids (Table 2) to form mixed bilayer systems of DOPC:DPPC, DOPC:DLPC, and DOPC:DTPC, all at a 75:25 molar ratio. Figure 9 presents representative Voronoi diagrams of the three CG lipid bilayer systems. Other relevant analyses for large CG systems of mixed lipid bilayers, such as examining the membrane surface area and looking at possible undulations in our CG systems, were not attempted here but could be done in future studies. The Voronoi cells associated with the secondary lipids do not exhibit significant clustering in large areas. Instead, they appear to be distributed randomly or form linear chainlike structures with only a few members. Since the primary and secondary lipids have identical headgroups, the formation of nanodomains in these binary systems was not expected, as the headgroups undergo electrostatic and dispersion interactions comparable to those found in pure bilayers.

**Figure 10:**
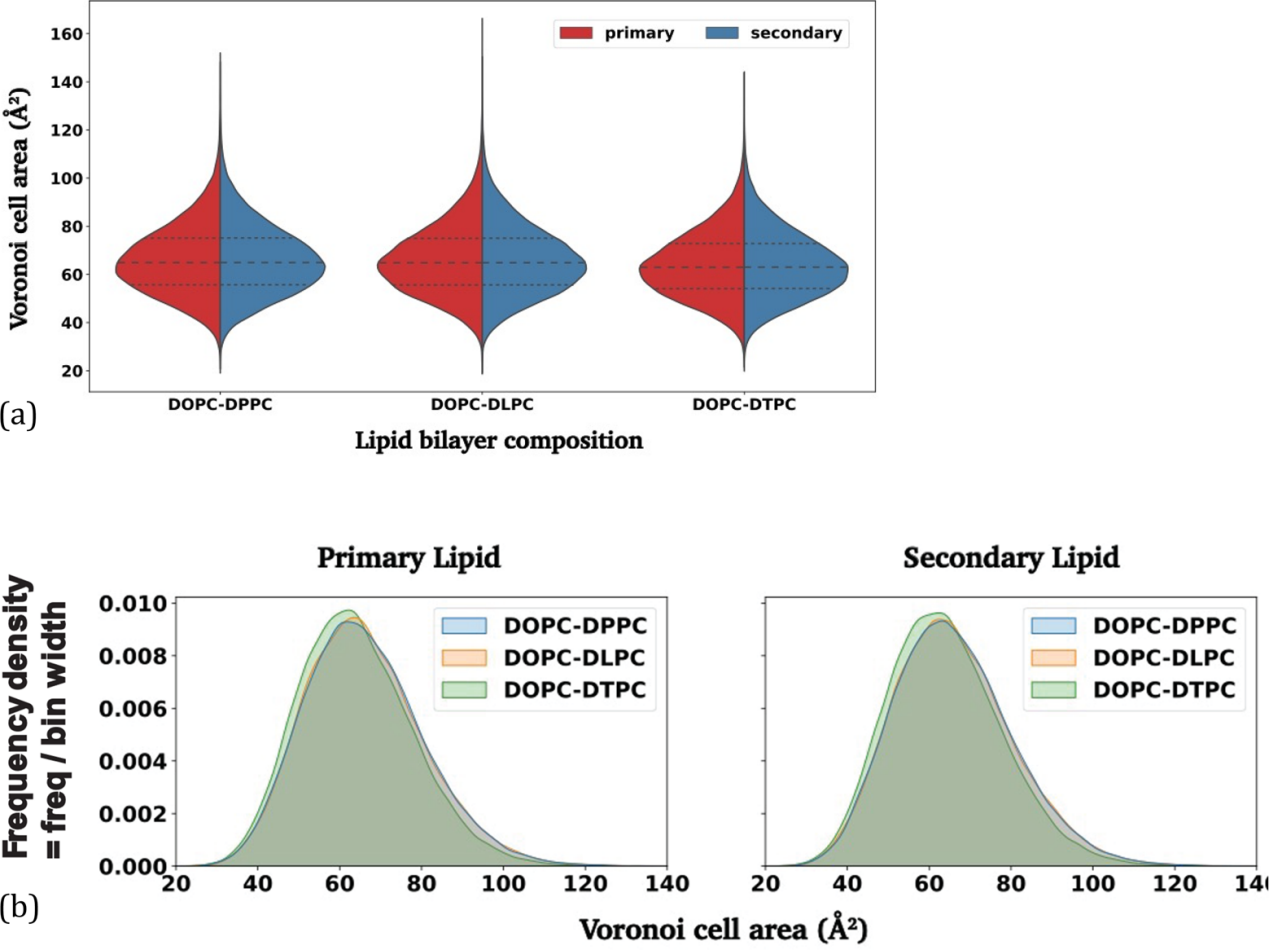
(a) Violin plots of the Voronoi cell area distribution of three bilayers with quartile dashed lines. The densities of DOPC and secondary lipids are colored in red and blue. (b) Histograms of Voronoi cell areas of primary lipid (DOPC), shown on the left, and secondary lipids (DTPC, DLPC, and DPPC), shown on the right, of three lipid bilayers.

Histograms illustrating the area distribution of Voronoi cells within the lipid bilayers can reveal information about the spatial organization of PO_4_ beads (Table 2), the uniformity or variability of the Voronoi cells, and the potential effects from different secondary lipids. In the violin plots shown in Figure 10(a), we compare the distributions of the Voronoi cell areas of primary and secondary lipids within the same bilayer system. Wider sections of the violin plots in the horizontal direction indicate larger probabilities that the primary and secondary lipids have a particular value of the Voronoi cell area. These results show that Voronoi cell areas are highly concentrated around ∼65 Å^2^ for all systems and that quartile results for both types of lipids are nearly indistinguishable across all systems. This outcome suggests that Voronoi cell areas and distributions are similar irrespective of the lipids’ acyl chain lengths. This observation was expected, given that our mixed bilayers consist of lipids with identical headgroups but varying acyl chain lengths, resulting in similar headgroup interactions in pure or mixed bilayers. However, when comparing histograms among different systems, Figure 10(b) reveals a slightly different Voronoi cell area distribution for primary and secondary lipids in DOPC-DTPC bilayers, compared to when DOPC is mixed with either DPPC or DLPC, which in contrast display nearly identical distributions. DOPC-DTPC systems exhibit peaks of slightly larger height, which are also shifted toward smaller Voronoi cell areas, compared to DOPC mixtures with DPPC or DLPC. Since DTPC possesses the shortest acyl chains among the three secondary lipids examined in our coarse-grained simulations (Table 2), DOPC and DTPC might pack more tightly in their mixed bilayer when compared to systems containing different secondary lipids.

To quantify the homogeneity (or inhomogeneity) of our lipid mixtures, we computed the percentage of mixed contacts using the following equation:^109^
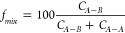
7The terms *C*_*A*−*A*_ and *C*_*A*–*B*_ represent the number of contacts between the same type of lipids and different lipids, respectively. These values describe the proportion of contacts between different lipids relative to the total number of contacts (same and different species). Two lipids are considered to be in contact if the distance between their phosphate group (PO_4_ beads) is smaller than or equal to 1.1 nm.^109^ The percentage of mixed contacts for our three CG Martini systems is shown in Table 3. These values suggest that all mixtures examined are homogeneous, as the numbers shown in Table 3 are comparable to the overall molar percentage of secondary lipids in our systems (25%). These results also show that the percentage of mixed contacts slightly increases as the secondary lipid has a shorter acyl chain length, although the increases are comparable to the uncertainties of our measurements. Following another previous study,^45^ we also determined the number and type of neighboring lipids around each lipid species as another way to analyze mixing in our CG systems. In these calculations, two lipids are considered neighbors if the distance between their first tail beads (labeled GL1 in Table 2) is smaller than or equal to 1.5 nm.^45^ These results are shown in Table 4 and again suggest that our CG lipid systems are homogeneous, as all local compositions of neighboring lipids are quite similar to the overall composition of our systems (75–25%).

**Table 3:** Percentage of mixed contacts in the CG systems

System	*f*_*mix*_
DOPC-DPPC	29.6 ± 0.5
DOPC-DLPC	29.9 ± 0.5
DOPC-DTPC	30.8 ± 0.5

**Table 4:** Number and Type of Neighboring Lipids in Our CG Systems^a^

Type of center lipid	Type of neighboring lipids	
	DOPC	DPPC
DOPC	6.73 ± 0.02 (67.4%)	3.26 ± 0.03 (32.6%)
DPPC	6.52 ± 0.06 (72.4%)	2.49 ± 0.06 (27.6%)
	DOPC	DLPC
DOPC	6.74 ± 0.02 (67.3%)	3.28 ± 0.02 (32.7%)
DLPC	6.55 ± 0.04 (72.9%)	2.44 ± 0.04 (27.1%)
	DOPC	DTPC
DOPC	6.87 ± 0.01 (66.6%)	3.45 ± 0.01 (33.4%)
DTPC	6.91 ± 0.03 (75.2%)	2.28 ± 0.04 (24.8%)

a For example, in a DOPC-DPPC system, a DOPC molecule is surrounded by an average of 6.73 DOPC and 3.26 DPPC lipid molecules, corresponding respectively to 67.4% and 32.6% of the average number of neighboring lipids.

## 4 Conclusions

This molecular simulation study provides initial insights into the complex relations between the lipid bilayer composition and lipid tail structure, with bilayer properties that might correlate with the elasticity and softness of the resulting liposomes. Using all-atom models, we examined lipid bilayers consisting of a binary mixture of the unsaturated, long-tailed phospholipids DOPC and DPMPC, combined with the shorter saturated lipids DHPC, DDPC, DLPC, and DMPC (Table 1), at varying lipid concentrations and surface tensions. Our results show that systems that have the largest examined concentrations of the shorter lipids DHPC and DDPC (25–35 mol %) mixed with DOPC or DPMPC have the smallest values of area compressibility moduli *K*_*A*_, ∼10% smaller than the values observed in pure DOPC or DPMPC bilayers. These observations indicate that these binary mixtures are the least rigid and most elastic bilayers among our examined systems, in agreement with micropipette aspiration measurements of the stretching moduli and lysis tension in liposomes with the same composition. Similar lipid bilayers consisting of binary mixtures of DOPC or DPMPC with a longer saturated lipid DLPC or DMPC in general have larger values of *K*_*A*_ compared to their DHPC or DDPC counterparts. Systems with large concentrations of DHPC or DDPC also have small values of area per lipid, as they have a more uneven *x*–*y* interface with water at any given value of surface tension, as compared to systems with smaller concentrations of DHPC or DDPC, systems having DLPC or DMPC, or pure DOPC or DPMPC bilayers. Furthermore, the tails in the primary lipids DOPC or DPMPC have smaller order parameters when they are in bilayers with larger amounts of DHPC or DDPC, which suggests that these binary systems are less ordered when compared to pure DOPC or DPMPC bilayers. Likewise, the order parameters of the tails of the secondary lipids in general follow the trend DMPC > DLPC > DDPC > DHPC, at any given mole fraction of the secondary lipid. Adding increasing concentrations of DHPC causes the terminal methyl groups in the tails of DOPC or DPMPC to wriggle more in the vertical direction, compared to systems with other secondary lipids or smaller concentrations of the secondary lipid.

Overall, these observations confirm our hypothesis that adding increasing concentrations of the short unsaturated lipid DHPC to DOPC or DPMPC bilayers would alter the lipid packing (Figure 1) and thus make the resulting liposomes more elastic and less rigid, compared to pure DOPC or DPMPC systems. Interestingly, no clear trends were observed in the lateral diffusion coefficients of the lipids as the concentration, type of secondary lipid, and surface tension were varied. Simulations of bilayers with larger *x*–*y* areas using coarse-grained models (∼3.75 larger *x* and *y* dimensions compared to all-atom simulations) suggest that our binary lipid mixtures do not form lipid nanodomains and confirm that lipid bilayers having a shorter-tail secondary lipid have smaller areas. Finally, we note that some of our mixtures seem to have *K*_*A*_ values that are up to 8.6% larger than the values observed in pure DOPC or DPMPC systems. At this moment, we are unable to elaborate on the possible reasons behind these increases in *K*_*A*_. Follow-up studies could focus on comparing area compressibility moduli and other properties such as bending moduli in these mixed bilayer systems, as determined from different computational methodologies that have been recently proposed.^46,108,109^

## Supporting Information Available

Lipid compositions studied and area compressibility modulus data (Tables S1–S2); area per lipid, bilayer thickness, and standard deviation propagation of vertical coordinates of headgroups at different surface tensions (Figures S1–S3); order parameter plots (Figures S4–S7); vertical spatial distribution of terminal methyl groups (Figure S8 and S9), with representative simulation snapshots (Figure S10); and lateral diffusivities at different surface tensions (Figure S11) (PDF)
