# Supplementary material for: Understanding the Mechanical Properties of Ultradeformable Liposomes Using Molecular Dynamics Simulations
Source: J Phys Chem B. 2023 Oct 25;127(44):9496–512. doi: 10.1021/acs.jpcb.3c04386 (PMC10641833; doi:10.1021/acs.jpcb.3c04386)
Supplement: Supplementary file 1 — jp3c04386_si_001.pdf [file jp3c04386_si_001.pdf]

Understanding the Mechanical Properties of Ultra-Deformable Liposome Membranes Using Molecular Dynamics Simulations

Supporting Information

Jiaming Xu,[1,†] Vyshnavi Karra, [1,†,‡] Danielle E. Large,[2] Debra T. Auguste[1,2,*] and Francisco R. Hung[1,*]
[1] Department of Chemical Engineering, Northeastern University, Boston, MA 02115
[2] Department of Bioengineering, Northeastern University, Boston, MA 02115

Table S1. AA and CG lipid compositions studied in this work. All simulations were performed at a temperature *T* = 298.15 K.

Bilayer composition	Molar ratio	Bilayer composition	Molar ratio
DOPC	100:0	DPMPC	100:0
DOPC:DHPC	95:5	DPMPC:DHPC	95:5
DOPC:DHPC	90:10	DPMPC:DHPC	90:10
DOPC:DHPC	85:15	DPMPC:DHPC	85:15
DOPC:DHPC	75:25	DPMPC:DHPC	75:25
DOPC:DHPC	65:35	DPMPC:DHPC	65:35
DOPC:DDPC	95:5	DPMPC:DDPC	95:5
DOPC:DDPC	85:15	DPMPC:DDPC	85:15
DOPC:DDPC	75:25	DPMPC:DDPC	75:25
DOPC:DDPC	65:35	DPMPC:DDPC	65:35
DOPC:DLPC	95:5	DPMPC:DLPC	95:5
DOPC:DLPC	85:15	DPMPC:DLPC	85:15
DOPC:DLPC	75:25	DPMPC:DLPC	75:25
DOPC:DLPC	65:35	DPMPC:DLPC	65:35
DOPC:DMPC	95:5	DPMPC:DMPC	95:5
DOPC:DMPC	85:15	DPMPC:DMPC	85:15
DOPC:DMPC	75:25	DPMPC:DMPC	75:25
DOPC:DMPC	65:35	DPMPC:DMPC	65:35
DOPC:DHPC (CG)	75:25	DPMPC:DHPC (CG)	75:25
DOPC:DDPC (CG)	75:25	DPMPC:DDPC (CG)	75:25

† Equal contribution
‡ Current address: Collaborative for Education Services, Department of Youth Services Education Initiative, Northampton, MA 01060
* Corresponding authors. E-mails: d.auguste@northeastern.edu, f.hung@northeastern.edu

Table S2. Area compressibility modulus K_A of our bilayer systems as determined from all-atom MD simulations. Molar percentages of primary and secondary lipid component are shown in parentheses. The columns labeled 'Difference' display the percentage change in K_A compared to the value obtained for the pure DOPC or DPMPC bilayers. A two-sample t-test was performed to assess the difference between each considered mixed system and their corresponding pure bilayer. The t-test was conducted at a 95% confidence level with degrees of freedom computed as $df = n_1 + n_2 - 2$. As all K_A values were determined from simulations at four values of surface tension, $n_1 = n_2 = 4$, giving $df = 6$. From the table of critical values of t for two-tailed tests[1], we obtain $t = 2.447$. From our t-test, p-values were also determined and reported below. Therefore, systems with absolute values of t-scores larger than 2.447, and with p-values smaller than 0.05 (marked in red below) indicate mixed bilayers with K_A values that are statistically different from the values observed in pure bilayer systems.

Bilayer composition	K_A (mN/m)	Difference	t-score	p-value	Bilayer composition	K_A (mN/m)	Difference	t-score	p-value
DOPC (100)	245.8 ± 9.9	-	-	-	DPMPC (100)	245.0 ± 6.4	-	-	-
DOPC-DHPC (95:5)	266.9 ± 6.4	8.59%	3.58	0.012	DPMPC-DHPC (95:5)	260.0 ± 3.5	6.13%	4.11	0.006
DOPC-DHPC (90:10)	251.5 ± 14.6	2.31%	0.65	0.540	DPMPC-DHPC (90:10)	232.9 ± 5.5	-4.93%	-2.87	0.028
DOPC-DHPC (85:15)	228.1 ± 9.7	-7.20%	-2.55	0.043	DPMPC-DHPC (85:15)	229.0 ± 5.8	-6.51%	-3.7	0.010
DOPC-DHPC (75:25)	220.3 ± 9.4	-10.40%	-3.74	0.010	DPMPC-DHPC (75:25)	229.9 ± 7.3	-6.16%	-3.11	0.021
DOPC-DHPC (65:35)	220.5 ± 3.8	-10.30%	-4.77	0.003	DPMPC-DHPC (65:35)	222.1 ± 2.0	-9.33%	-6.83	0.000
DOPC-DDPC (95:5)	245.1 ± 10.7	-0.27%	-0.1	0.924	DPMPC-DDPC (95:5)	245.0 ± 4.9	0.03%	0	1.000
DOPC-DDPC (85:15)	224.8 ± 12.7	-8.53%	-2.61	0.040	DPMPC-DDPC (85:15)	243.8 ± 6.5	-0.47%	-0.26	0.804
DOPC-DDPC (75:25)	224.3 ± 6.1	-8.77%	-3.7	0.010	DPMPC-DDPC (75:25)	238.9 ± 4.0	-2.49%	-1.62	0.156
DOPC-DDPC (65:35)	220.5 ± 11.0	-10.30%	-3.42	0.014	DPMPC-DDPC (65:35)	223.2 ± 5.8	-8.88%	-5.05	0.002
DOPC-DLPC (95:5)	236.1 ± 10.4	-3.95%	-1.35	0.226	DPMPC-DLPC (95:5)	258.8 ± 6.4	5.65%	3.05	0.023
DOPC-DLPC (85:15)	233.6 ± 4.5	-4.97%	-2.24	0.066	DPMPC-DLPC (85:15)	251.9 ± 6.7	2.83%	1.49	0.187
DOPC-DLPC (75:25)	241.6 ± 5.5	-1.71%	-0.74	0.487	DPMPC-DLPC (75:25)	240.7 ± 4.2	-1.74%	-1.12	0.306
DOPC-DLPC (65:35)	229.4 ± 6.5	-6.68%	-2.77	0.032	DPMPC-DLPC (65:35)	246.4 ± 6.1	0.58%	0.32	0.760
DOPC-DMPC (95:5)	249.1 ± 3.0	1.35%	0.64	0.546	DPMPC-DMPC (95:5)	254.1 ± 7.5	3.73%	1.85	0.114
DOPC-DMPC (85:15)	226.8 ± 4.5	-7.73%	-3.49	0.013	DPMPC-DMPC (85:15)	240.0 ± 2.6	-2.03%	-1.45	0.197
DOPC-DMPC (75:25)	231.2 ± 8.7	-5.94%	-2.22	0.068	DPMPC-DMPC (75:25)	241.9 ± 3.3	-1.25%	-0.86	0.423
DOPC-DMPC (65:35)	226.1 ± 10.7	-8.02%	-2.7	0.036	DPMPC-DMPC (65:35)	247.8 ± 8.5	1.15%	0.53	0.615

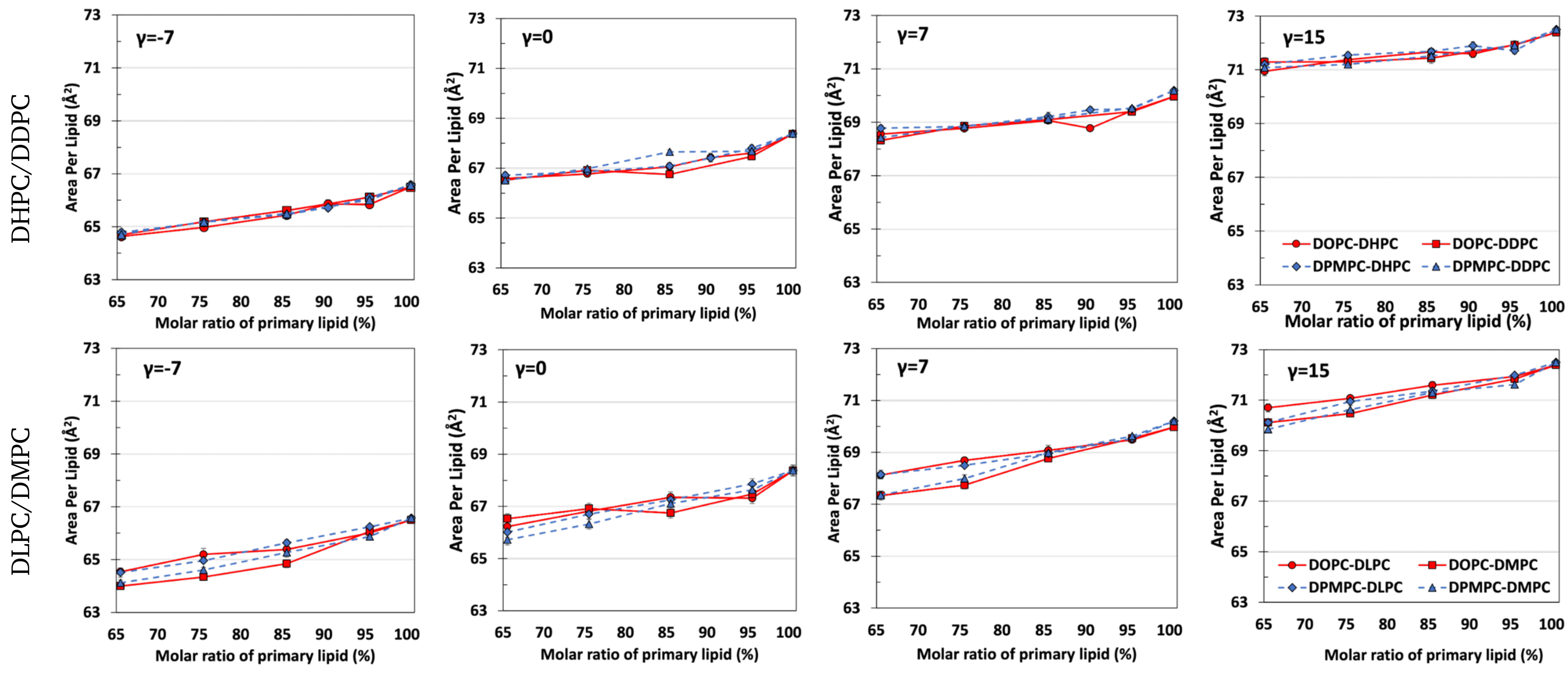

Figure S1: Area per lipid of different bilayers as a function of the molar ratio of primary lipid and at different values of surface tension γ (in mN/m). In all figures, DOPC-based bilayers are shown in red and DPMPC-based bilayers in blue. Top row results corresponds to mixed bilayers with DHPC or DDPC, wereas bottom row results corresponds to bilayers having DLPC or DMPC as secondary lipids.

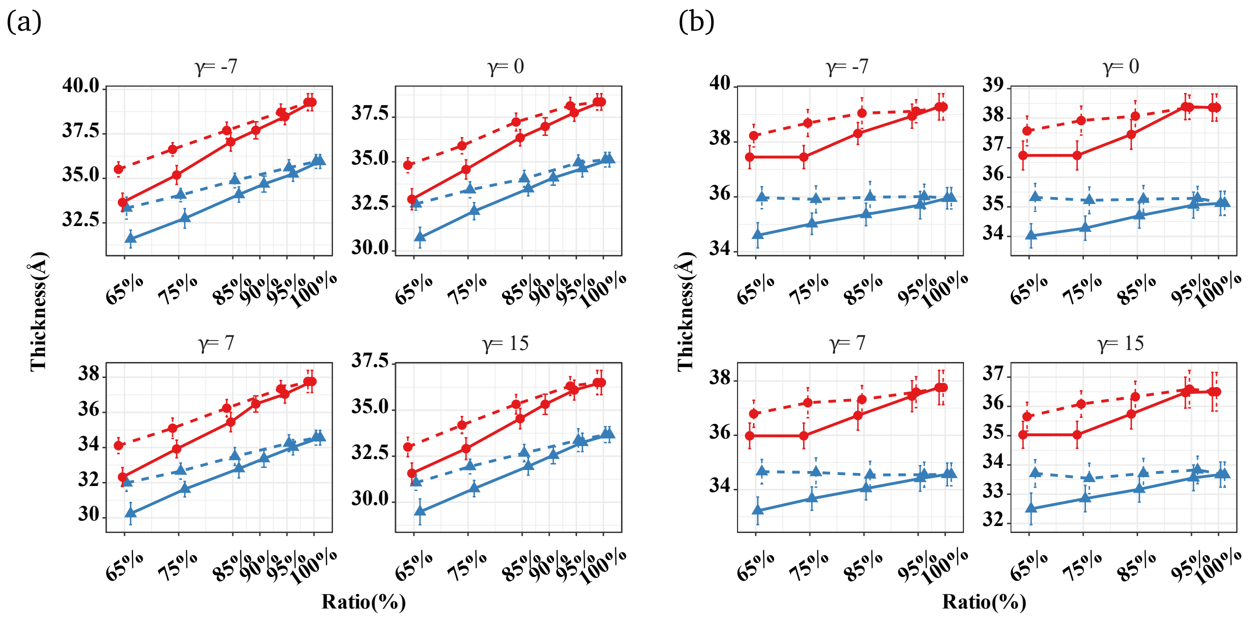

Figure S2: Lipid bilayer thickness as a function of molar ratio of primary lipid and at different values of surface tension γ (in mN/m). Systems are shown as follows: DOPC (red lines with circles), DPMPC (blue lines with triangles), DHPC (solid lines in (a)), DDPC (dashed lines in (a)), DLPC (solid lines in (b)), and DMPC (dashed lines in (b)). Although all the binary mixtures depicted had the same compositions (65, 75, 85, 95 and 100 mole% of the long unsaturated lipid, and for some systems 90%), all data points shown in the figure were slightly displaced horizontally around these compositions for ease of visualization. Simulations at non-zero surface tensions might capture phenomena relevant to situations where deformable liposomes squeeze through fibrous tissue.

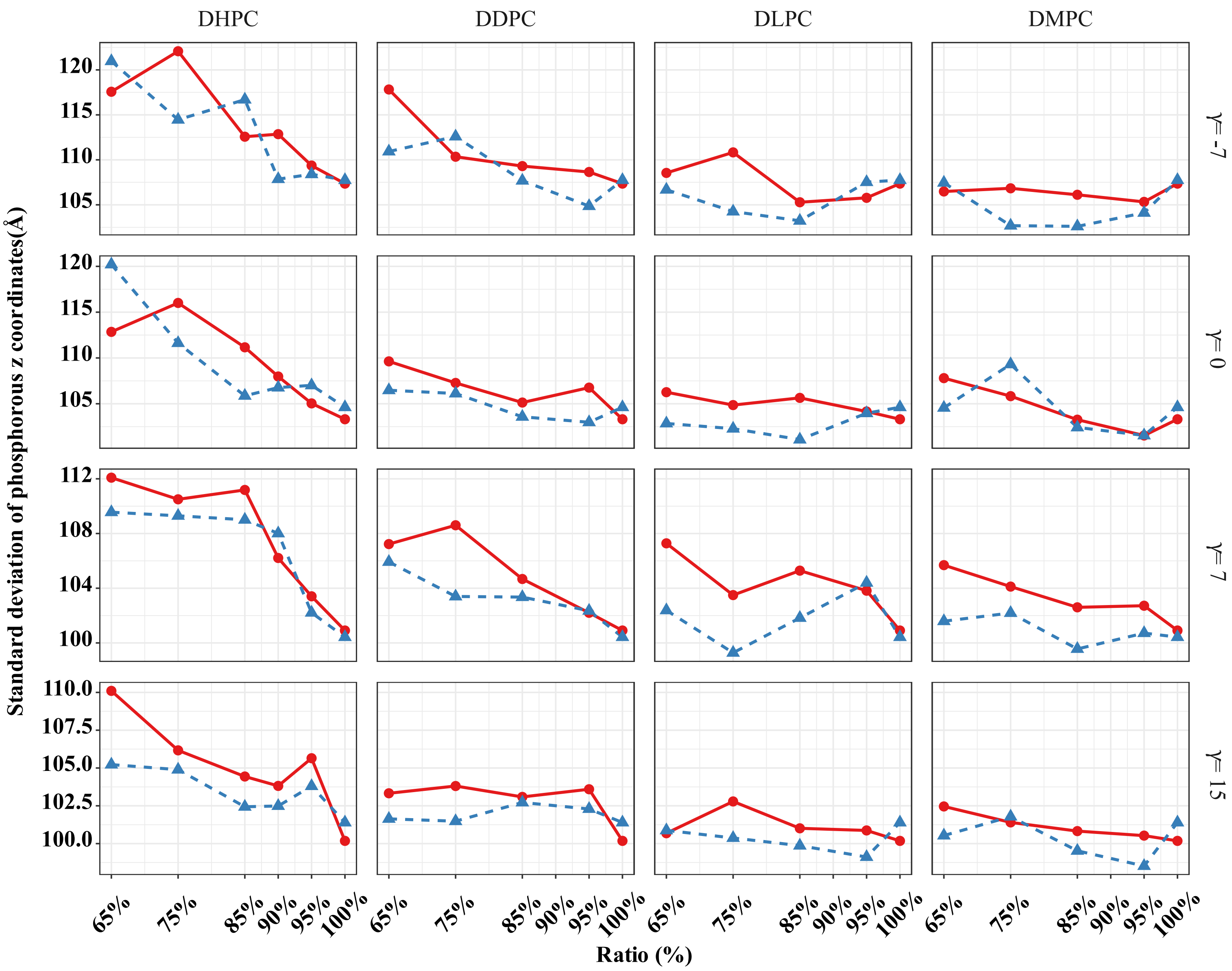

Figure S3. Sum of the standard deviations (distances, in Å) from the average z-coordinate of phosphorous atoms, as indicators of the surface unevenness of the bilayer structure. These values were calculated over 500 random simulation frames (see text for details of how these summed distances were computed), as a function of molar ratio of long tail lipids at the surface tension values indicated at the right of each row (in mN/m). Secondary lipids considered are indicated by the top labels over each plot. Red solid lines with circles = DOPC; blue dashed lines with triangles = DPMPC. Simulations at non-zero surface tensions might capture phenomena relevant to situations where deformable liposomes squeeze through fibrous tissue.

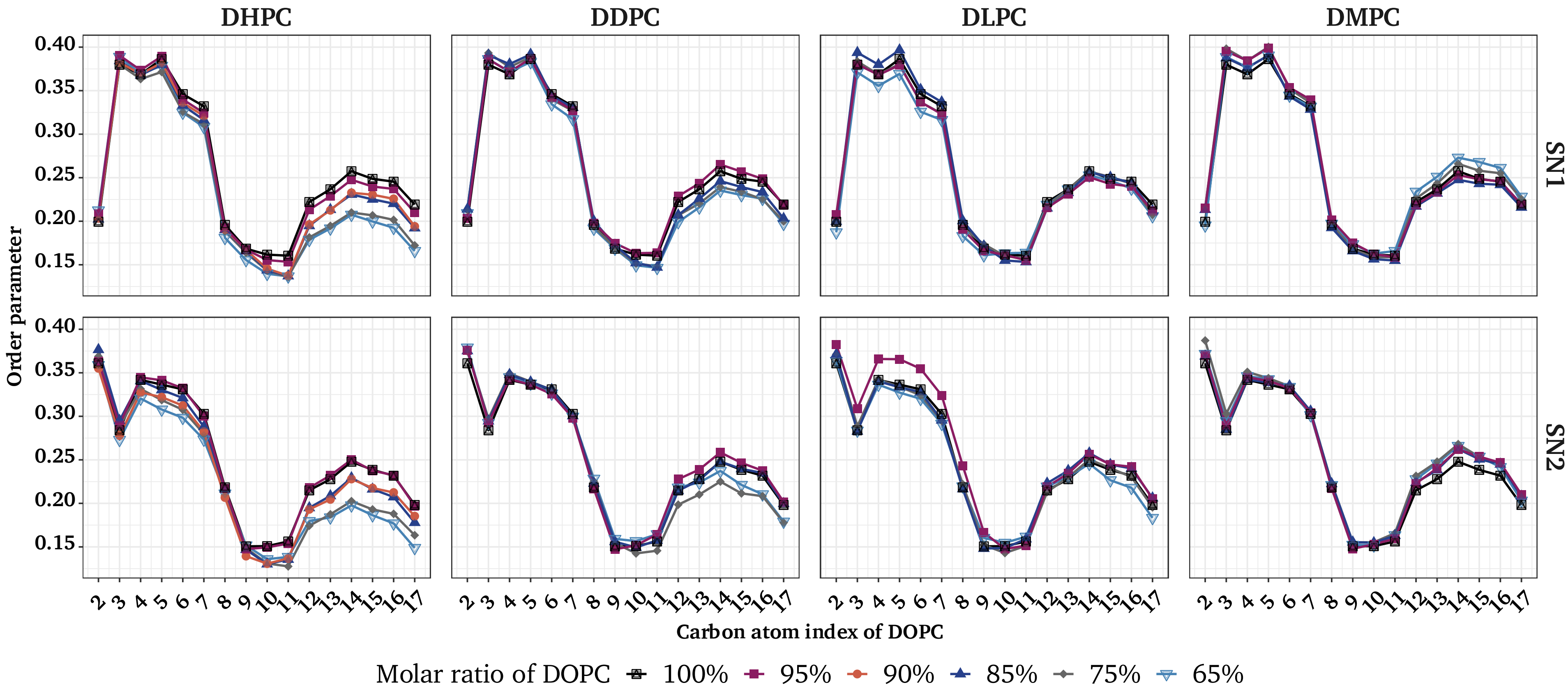

Figure S4: Order parameters in the two acyl chains SN1 and SN2 (rows) of the primary lipid in DOPC-based bilayers, for different secondary lipids (columns) at different mole fractions (lines in each plot). All results were obtained at a surface tension $\gamma = 0$ mN/m.

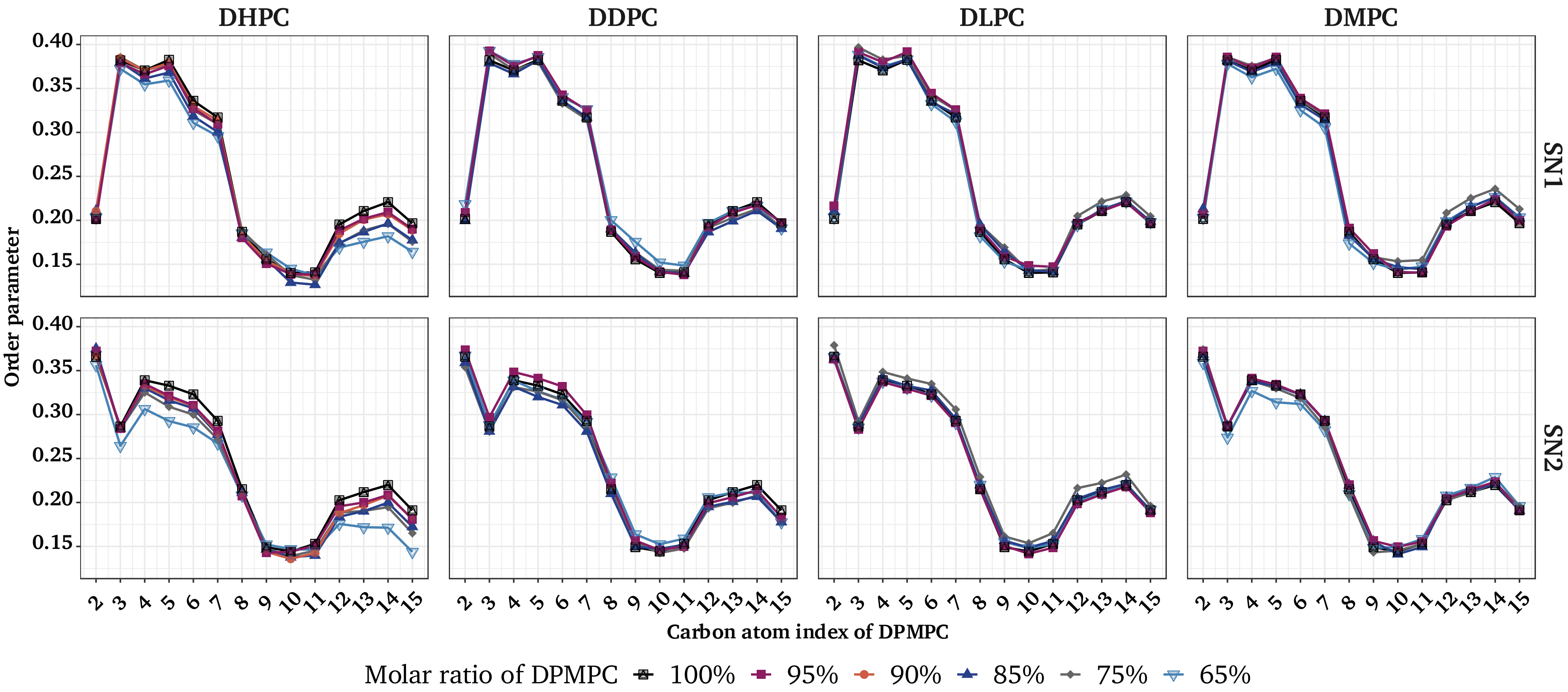

Figure S5: Order parameters in the two acyl chains SN1 and SN2 (rows) of the primary lipid in DPMPC-based bilayers, for different secondary lipids (columns) at different mole fractions (lines in each plot). All results were obtained at a surface tension $\gamma = 0$ mN/m.

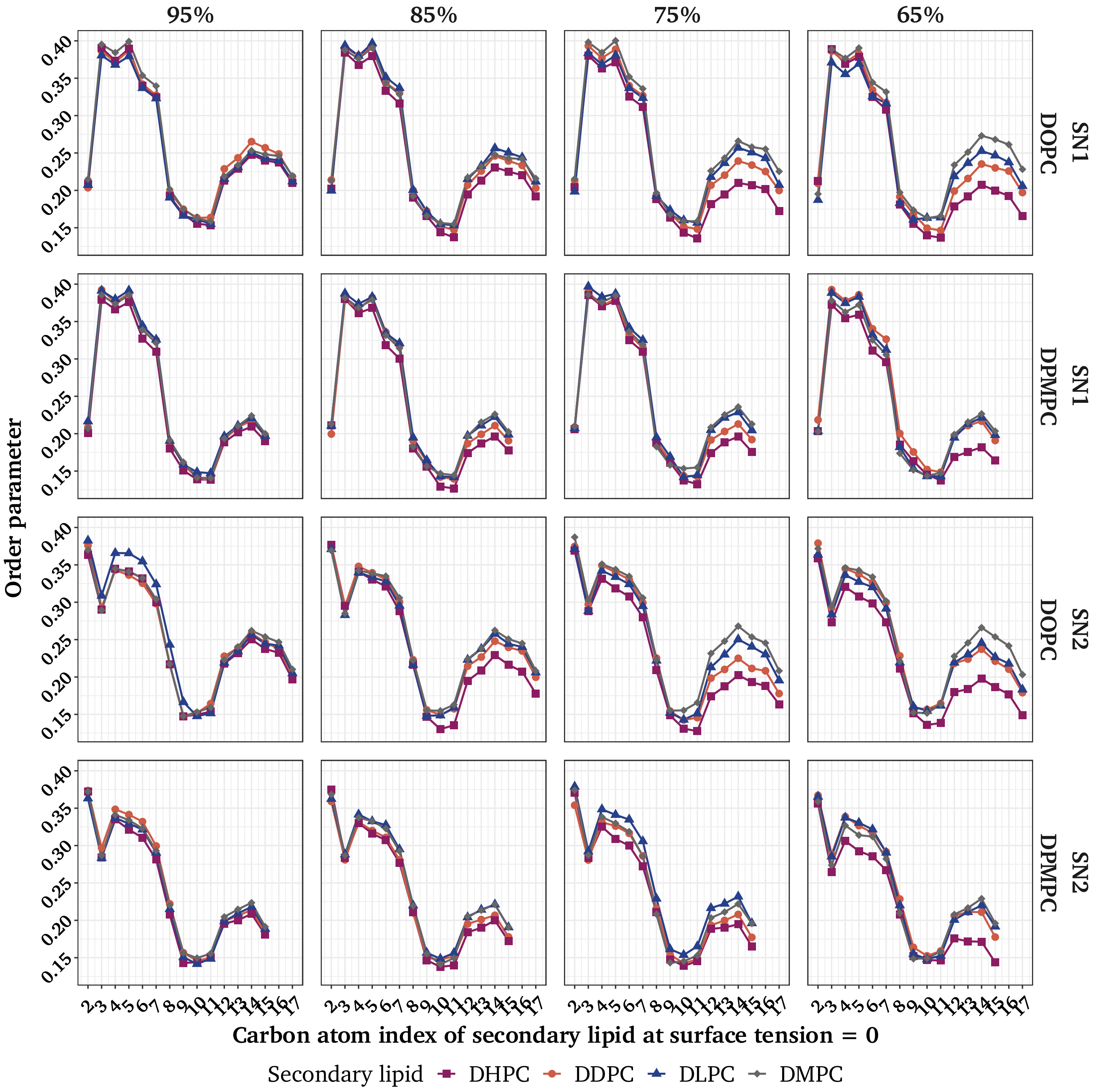

Figure S6: Order parameters in the two acyl chains SN1 and SN2 of the primary lipid in DOPC- and DPMPC-based bilayers (different rows), at fixed compositions (different columns), where the lines in each plot represent the different secondary lipids. These results are the same as those shown in Figures S4 and S5, just represented in a different way. All results were obtained at a surface tension $\gamma = 0$ mN/m.

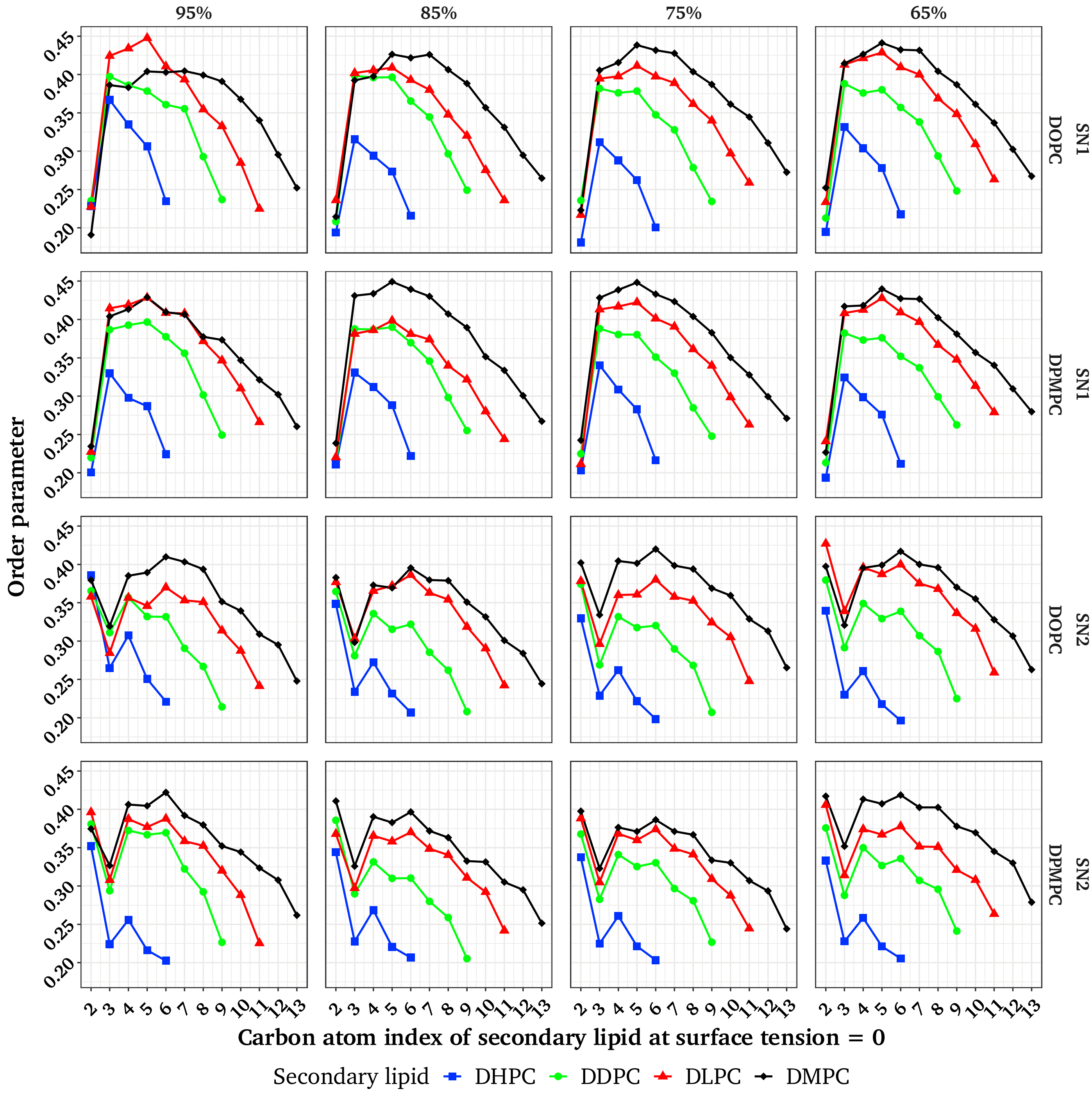

Figure S7: Order parameter in the acyl tails of the different secondary lipids. Each column shows results of same primary lipid ratio, first two rows show results of SN1, and last two rows show SN2 acyl chain. All results were obtained at a surface tension $\gamma = 0$ mN/m.

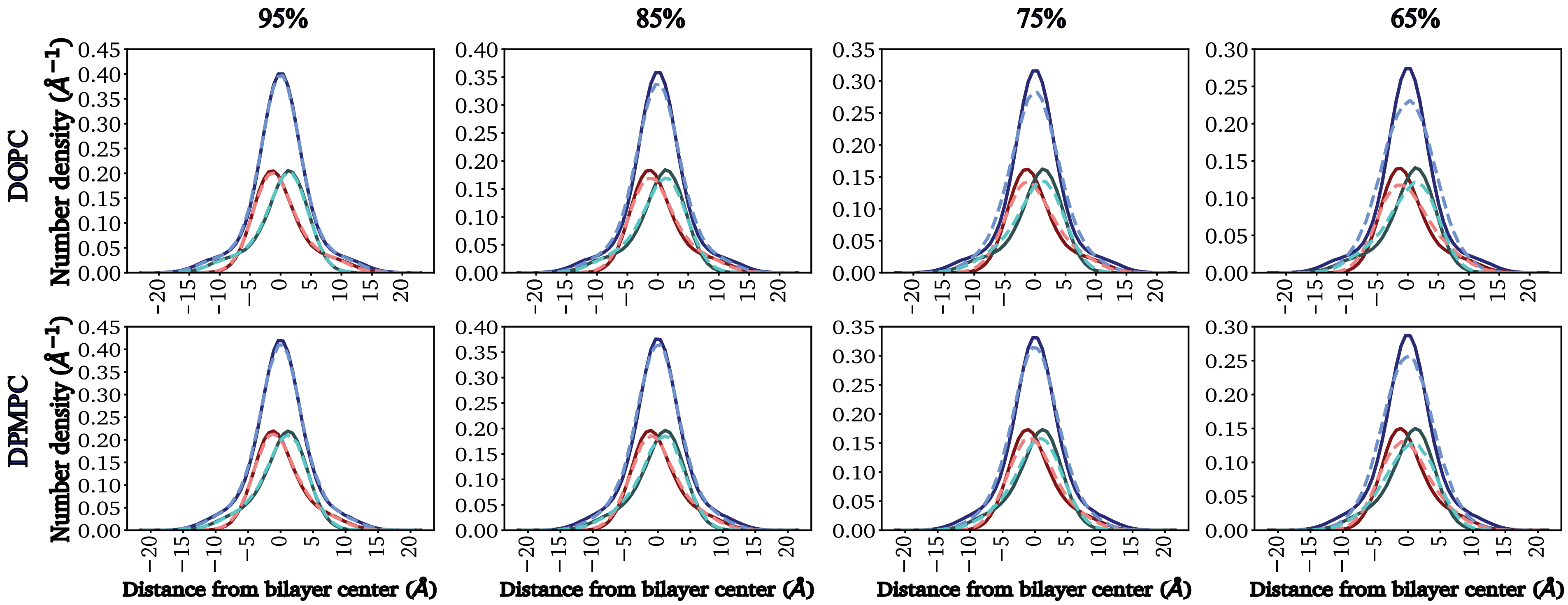

Figure S8: Vertical spatial distribution of terminal methyl groups (TMGs) in primary lipids, for systems DOPC-DHPC (top row) or DPMPC-DHPC (bottom row) at a surface tension of $\gamma = 0$ mN/m. Results observed in systems with different compositions are shown in the vertical columns. Results for mixed bilayers (dashed, light-colored lines) are compared against results for pure bilayers (solid, dark-colored lines). Dark/light red, dark/light green and dark/light blue represent the TMG distribution for leaflet 1, leaflet 2, and both leaflets, respectively. Simulations at non-zero surface tensions might capture phenomena relevant to situations where deformable liposomes squeeze through fibrous tissue.

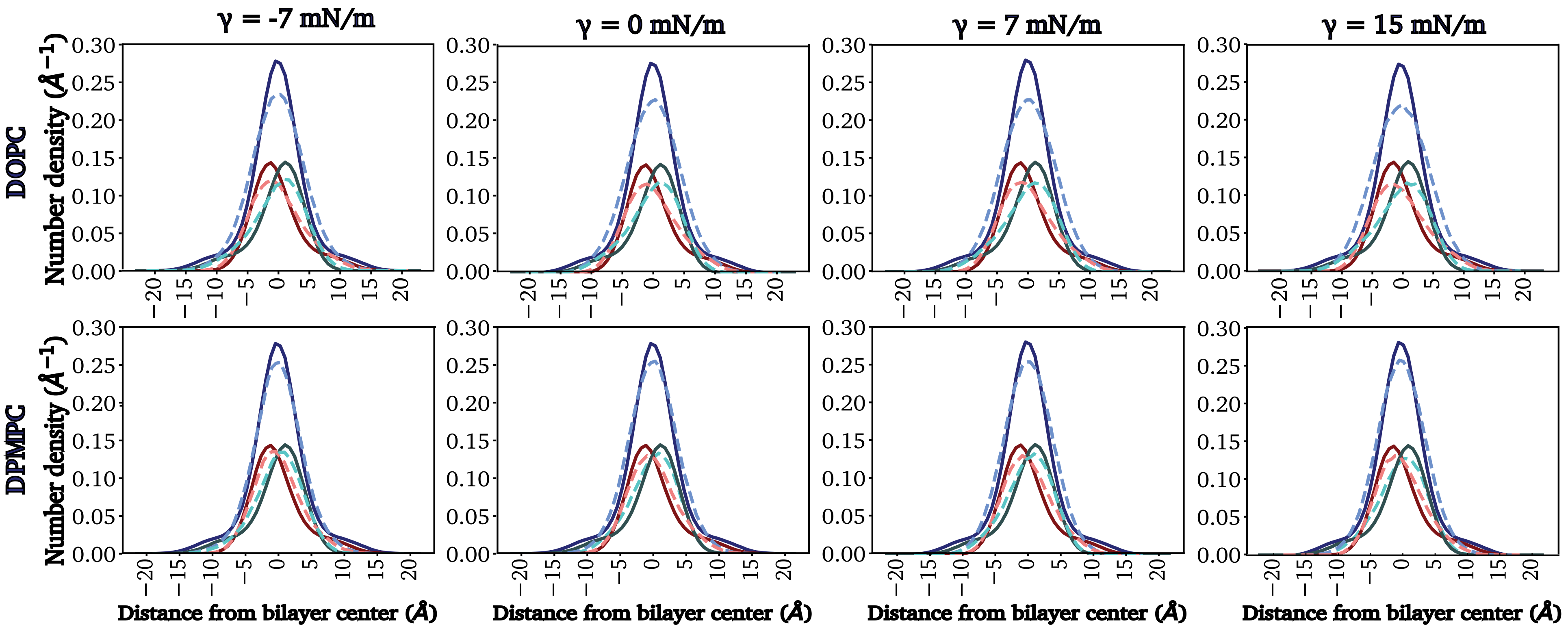

Figure S9: Vertical spatial distribution of terminal methyl groups (TMGs) in primary lipids, for systems DOPC-DHPC (65-35) (top row) or DPMPC-DHPC (65-35) (bottom row). Results observed in systems with different surface tensions are shown in the vertical columns. Results for mixed bilayers (dashed, light-colored lines) are compared against results for pure bilayers (solid, dark-colored lines). Dark/light red, dark/light green and dark/light blue represent the TMG distribution for leaflet 1, leaflet 2, and both leaflets, respectively. Simulations at non-zero surface tensions might capture phenomena relevant to situations where deformable liposomes squeeze through fibrous tissue.

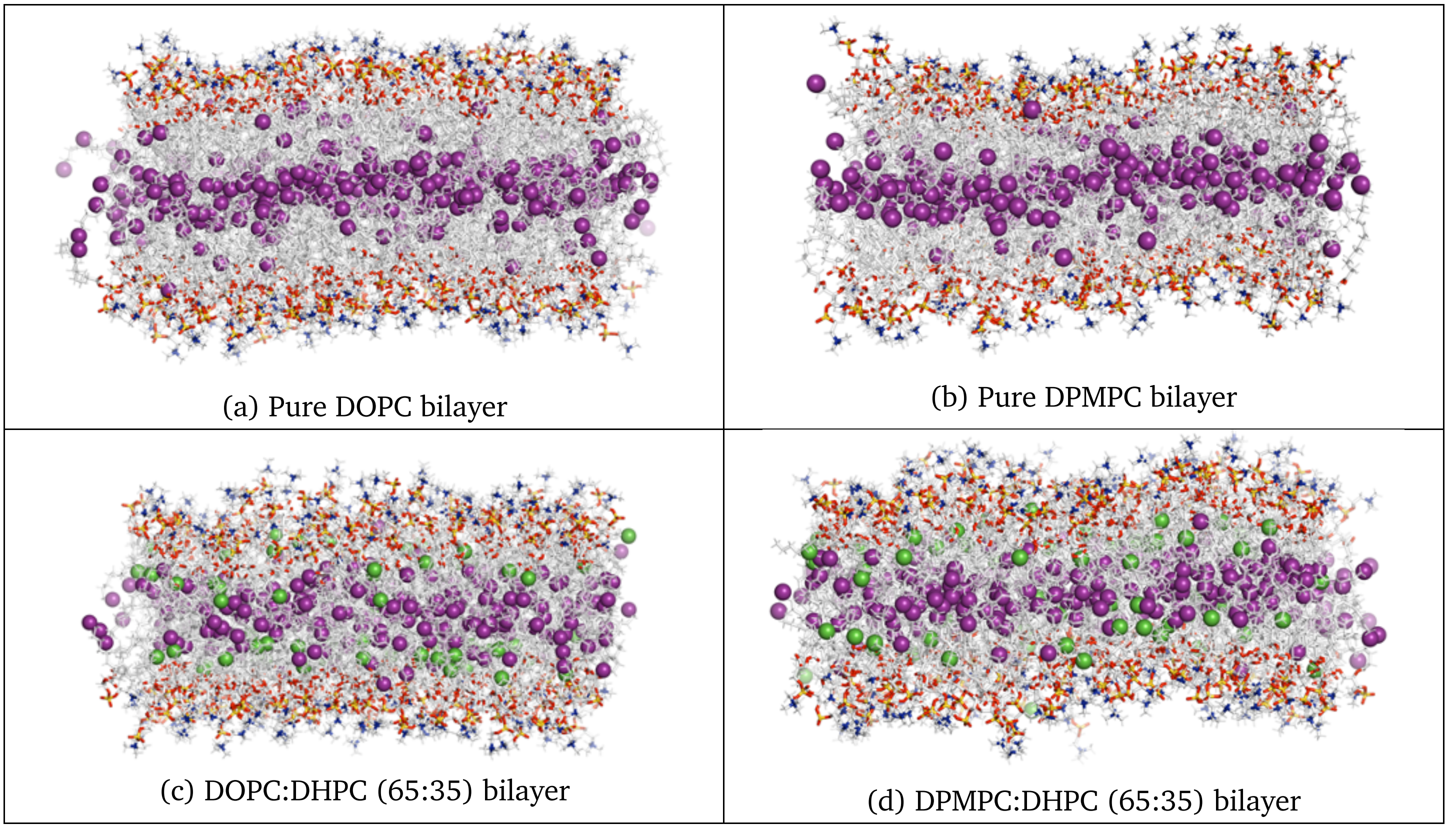

Figure S10. Representative simulation snapshots of AA MD simulations of (a) pure DOPC bilayer, (b) pure DPMPC bilayer, (c) DOPC:DHPC (65:35) bilayer, and (d) DPMPC:DHPC (65:35) bilayer. Terminal methyl groups are shown as spheres (purple = primary lipids; green = secondary lipids); the other atoms in lipid molecules and colored according to atom types (blue = nitrogen; red = oxygen; gold = phosphorous; white = carbon and hydrogen). Water and ions not shown for clarity.

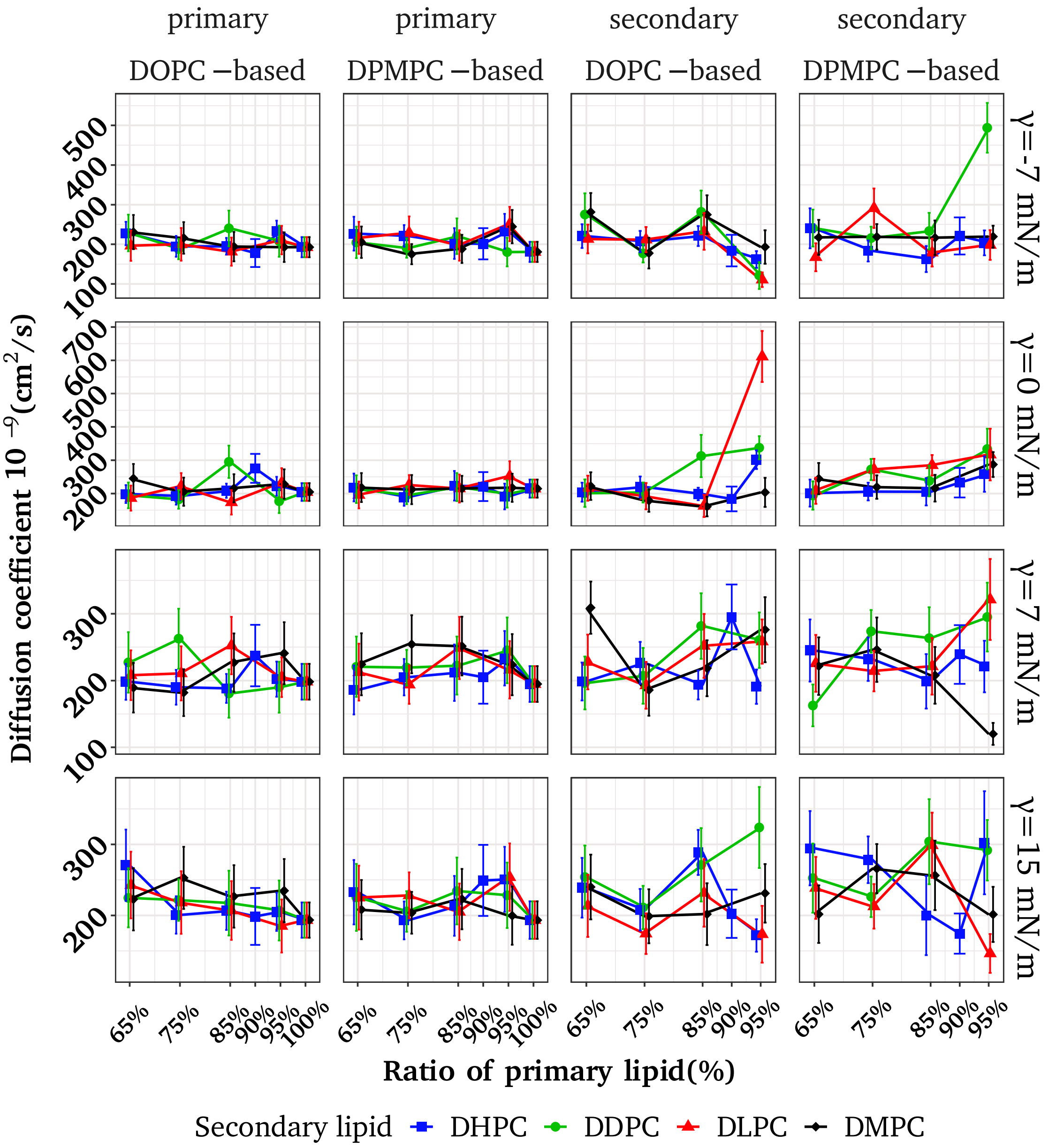

Figure S11: Diffusion coefficients of lipids in all systems. The first two columns show results for the primary lipids (DOPC or DPMPC), whereas the last two columns depict the diffusivities of the secondary lipids. Each row shows results at different values of surface tension γ (in mN/m), as shown in the label on the right. Lines of different colors represent the different secondary lipid, as indicated in the legend. Systems with 10 secondary lipids gave a considerably large diffusion coefficient due to sample size, like DLPC in 95%DOPC-DLPC bilayer and DDPC in 95% DPMPC-DDPC bilayer. Although all the binary mixtures depicted had the same compositions (65, 75, 85, 95 and 100 mole% of the long unsaturated lipid, and for some systems 90%), all data points shown in the figure were slightly displaced horizontally around these compositions for ease of visualization. Simulations at non-zero surface tensions might capture phenomena relevant to situations where deformable liposomes squeeze through fibrous tissue.

References

(1) Shaun, T. *Student's t Table (Free Download) | Guide & Examples*. https://www.scribbr.com/statistics/students-t-table/ (accessed 2023-09-17).
